# c-Jun N-terminal kinase (JNK)-dependent internalization and Rab5-dependent endocytic sorting mediate long-distance retrograde neuronal death induced by axonal BDNF-p75 signaling

**DOI:** 10.1038/s41598-019-42420-6

**Published:** 2019-04-15

**Authors:** C. A. Escudero, C. Cabeza, G. Moya-Alvarado, M. T. Maloney, C. M. Flores, C. Wu, F. A. Court, W. C. Mobley, F. C. Bronfman

**Affiliations:** 1 Center for Aging and Regeneration (CARE UC) and Department of Physiology, Faculty of Biological Sciences, Pontificia Universidad Católica de Chile, Santiago, Chile; 2 Department of Neurology and Neurological Sciences, Stanford University School of Medicine, Stanford, CA USA; 3 Center for Integrative Biology, Faculty of Sciences, Universidad Mayor, Santiago, Chile and FONDAP Center for Geroscience, Brain Health and Metabolism, Santiago, Chile; 4 Department of Neurosciences, University of California, San Diego, La Jolla, California, 92093 USA; 5 Present Address: Center of Cellular and Integrative Physiology, Faculty of Medicine, Clínica Alemana, Universidad del Desarrollo, Santiago, Chile

## Abstract

During the development of the sympathetic nervous system, signals from tropomyosin-related kinase receptors (Trks) and p75 neurotrophin receptors (p75) compete to regulate survival and connectivity. During this process, nerve growth factor (NGF)- TrkA signaling in axons communicates NGF-mediated trophic responses in signaling endosomes. Whether axonal p75 signaling contributes to neuronal death and how signaling endosomes contribute to p75 signaling has not been established. Using compartmentalized sympathetic neuronal cultures (CSCGs) as a model, we observed that the addition of BDNF to axons increased the transport of p75 and induced death of sympathetic neurons in a dynein-dependent manner. In cell bodies, internalization of p75 required the activity of JNK, a downstream kinase mediating p75 death signaling in neurons. Additionally, the activity of Rab5, the key GTPase regulating early endosomes, was required for p75 death signaling. In axons, JNK and Rab5 were required for retrograde transport and death signaling mediated by axonal BDNF-p75 in CSCGs. JNK was also required for the proper axonal transport of p75-positive endosomes. Thus, our findings provide evidence that the activation of JNK by p75 in cell bodies and axons is required for internalization to a Rab5-positive signaling endosome and the further propagation of p75-dependent neuronal death signals.

## Introduction

Neurotrophins are a well-known family of proteins comprising nerve growth factor (NGF), brain-derived neurotrophic factor (BDNF), neurotrophin-3 (NT-3) and neurotrophin-4 (NT-4). When bound to their respective cognate receptors, the tropomyosin-related kinases or “Trks” (TrkA, TrkB and TrkC) and the p75 neurotrophin receptor (p75), neurotrophins regulate many aspects of neuron structure and function, including survival, differentiation and the development and maintenance of neuronal circuits.

p75 triggers different biological outcomes than those mediated by Trks, including apoptosis and axon degeneration, with actions dependent on its expression level and/or its association with coreceptors at the plasma membrane^1–3^. The p75 protein is a multifunctional receptor that induces neuronal death in the developing and injured mature nervous system^4^. The pathways responsible for the production of ceramides, regulation of RhoA activity, activation of c-jun N-terminal activated kinase (JNK), translocation of NFκB^5–7^ and nuclear accumulation of neurotrophin receptor-interacting factor (NRIF) are among those activated by p75^8^. In turn, JNK and NRIF have been shown to regulate p75-mediated apoptosis in sympathetic neurons^8–11^.

Studies aiming to decipher the mechanisms that underlie the actions of Trks and p75 and how they either collaborate or compete to create neuronal phenotypes are actively pursued. Given the polarity of neurons and the extraordinary challenges posed by the long -distance transport of cellular signals, an important focus is how axonal signals are communicated to the cell body. A well-defined cell culture model for axon to soma communication employs sympathetic neurons from the superior cervical ganglia (SCGs). SCGs express two types of neurotrophin receptors, the NGF-specific receptor tyrosine kinase TrkA and the p75 receptor (which binds all neurotrophins). Target-derived NGF interacts with axonal TrkA receptors to induce internalization by creating a signaling endosome containing the NGF/activated TrkA complex, which is retrogradely trafficked to the soma. Therein, the NGF/activated TrkA complex triggers changes in gene expression and other cellular events required for neuronal survival and differentiation. Remarkably, the NGF/activated TrkA signal competes with and silences a cell death signal induced by the binding of BDNF to p75^12^. Through this latter pathway, p75 signaling appears to impact the developing sympathetic nervous system by reducing the number of neurons and target innervation^13,14^. The subcellular loci within neurons in which BDNF/p75 signals are important for the phenotypes produced. Thus, while BDNF/p75 signals generated within axons induce axonal pruning^14^, signals at the cell body induce neuronal death^15^. Based on accumulating evidence, target-derived BDNF induces cell death in developing SCGs^16,17^. However, the cellular mechanisms and the role played in neuronal degeneration by endosomes carrying p75 signaling in axons have not been addressed.

One possibility is that BDNF binding to axonal p75 receptors creates endosomes that retrogradely transport signals that induce the death of developing SCG neurons. As shown in previous studies, p75 mediates retrograde stress signaling by proneurotrophins, and signaling endosomes purified from the sciatic nerve axoplasm of a mouse model of amyotrophic lateral sclerosis (ALS) contain both p75 and JNK^17,18^. Additionally, p75 is retrogradely transported within axons of a variety of neuronal subtypes^19,20^. Here, we examined axonal p75 signaling using compartmentalized sympathetic neuronal cultures (CSCGs) and showed that axonally applied BDNF induced retrograde transport of p75 and apoptosis. We showed that delivery of the apoptotic signal from axons to cell bodies requires the activity of the monomeric GTPase Rab5 and dynein. Also, inhibition of Rab5 activity reduces the levels of cleaved-caspase 3 and the nuclear accumulation of NRIF in neuronal cell bodies of noncompartmentalized cultures. In our examination of the signaling pathways implicated in apoptotic signaling, BDNF activated JNK in axons, and JNK activity was required for the internalization and retrograde axonal transport of p75. Additionally, JNK inhibition reduced axonal BDNF/p75-mediated neuronal apoptosis. Our results provide evidence that the activation of JNK induced by the binding of BDNF to p75 on axons increases the internalization of p75 into Rab5-positive organelles, an event that is required to propagate p75-induced death signaling to cell bodies. These findings are evidence that Rab5-positive signaling endosomes are required to propagate p75 death signaling.

## Results

### BDNF-dependent retrograde transport of p75 and subsequent death signaling in CSCG neurons require dynein

**Figure 1:**
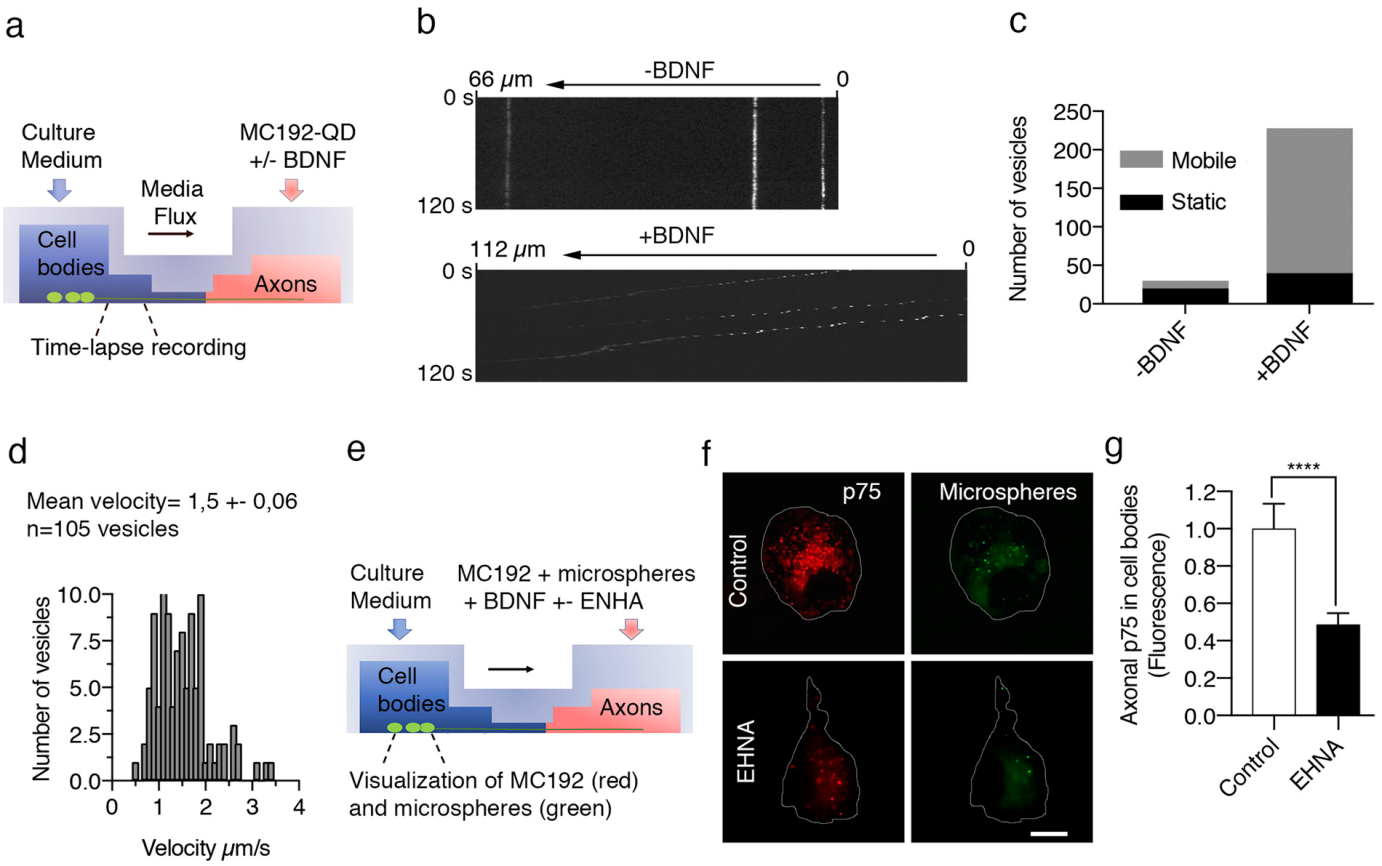
Axonal p75 is retrogradely transported in a ligand- and dynein-dependent manner. (**a**) Illustration of the design of the experiment used to study retrograde transport and signaling in sympathetic neurons. (**b**) Kymographs of axonal p75 movement in response to BDNF. Compartmentalized sympathetic neuron cultures were treated with BDNF (200 ng/mL) and MC192-QD, which labeled the extracellular domain of p75, for 4 hours at 37 °C in the axon compartment before imaging. Upper panels show kymographs of retrograde p75 movement in the absence (−) and presence (+) of BDNF in the axon compartment. (**c**) The graph indicates the proportion of vesicles without (black) or with (gray) movement when axon chambers were treated without (−) or with (+) BDNF. Data were obtained from videos captured in three independent experiments. (**d**) Histogram showing the distribution of velocities measured from 105 moving vesicles. The mean velocity of p75 vesicles undergoing retrograde transport in the presence of BDNF was approximately 1.5 µm/second. (**e**) Illustration of the design of the experiment used to study the retrograde transport of p75 and its dependence on dynein. (**f**) Visualization of axonally labeled p75 in the cell bodies of compartmentalized sympathetic neurons. The axons of compartmentalized neuronal cultures were treated with BDNF and MC192-Alexa Fluor 594 (to label p75, red) and microspheres labeled with Alexa Fluor 488 (to label compartmentalized neurons, green) for 16 hours at 37 °C in the absence (control) or presence of the dynein inhibitor EHNA (erythro-9-(2-hydroxy-3-nonyl). Scale bar, 10 μm. (**g**) Levels of axonally labeled p75 in the absence (control) or presence of EHNA. Data from 120 cells in four different compartmentalized chambers were quantified. Microspheres labeled with Alexa Fluor 488 were added 24 hours before the BDNF treatment and maintained in the culture throughout the experiment. Statistically significant differences were analyzed using a two-tailed Mann-Whitney test. ****p < 0,0001.

The retrograde transport and the subsequent neuronal death signaling induced by the activation of p75 in axons has increasingly been documented^16,17,20–22^. However, few studies have examined the cellular and molecular mechanisms regulating this process. We incubated CSCGs neurons with 12.5 mM KCl, as described in the methods, Fig. 1a and elsewhere^9,16,23^, to explore a physiological role for p75 axonal signaling in the absence of TrkA signaling and to avoid cell death induced by neurotrophin withdrawal. Supplementary Figure 1 summarizes the design of the experiments used to study the retrograde transport and death signaling mediated by BDNF/p75 in this study. We first assessed whether p75 was retrogradely transported in a ligand-dependent manner by applying the MC192 monoclonal antibody against the extracellular domain of p75 to axons of CSCG cultures. This antibody does not interfere with the binding of neurotrophins to p75 and was used to track the internalization and postendocytic trafficking of p75 in live cells^24^. The MC192 antibody was coupled to quantum dots (QDots) to study the retrograde transport of p75 under the microscope in real time. The presence of BDNF substantially increased the retrograde transport and number of p75-positive vesicles in axons (Fig. 1b,c and movies 1 and 2 in the Supplementary Information). The p75-positive vesicles exhibited continuous, unidirectional retrograde transport (Fig. 1d), with a mean velocity of approximately 1.5 μm per second (Fig. 1D), a value consistent with fast axonal transport^25^ and the retrograde axonal endosomal trafficking of NGF and BDNF in sensory and central neurons, respectively^26,27^. Consistent with these findings, we observed colocalization of BDNF and p75 in most but not all p75-positive vesicles in MC192- and BDNF-treated axons using double immunofluorescence staining (Supplementary Figure 2). Thus, p75 is retrogradely transported with its ligand, and, consistent with our previous results and the results from other studies, a basal level of p75 internalization and transport occurs in the absence of ligand^19,24,28^.

To address the role of axonal dynein, we treated the axonal compartment with EHNA, a compound that specifically blocks the ATPase activity of dynein, but not kinesins or myosins^29^ as shown in Fig. 1e. The addition of EHNA to the axonal compartment significantly reduced the retrograde transport of p75 induced by BDNF (Fig. 1f,g).

**Figure 2:**
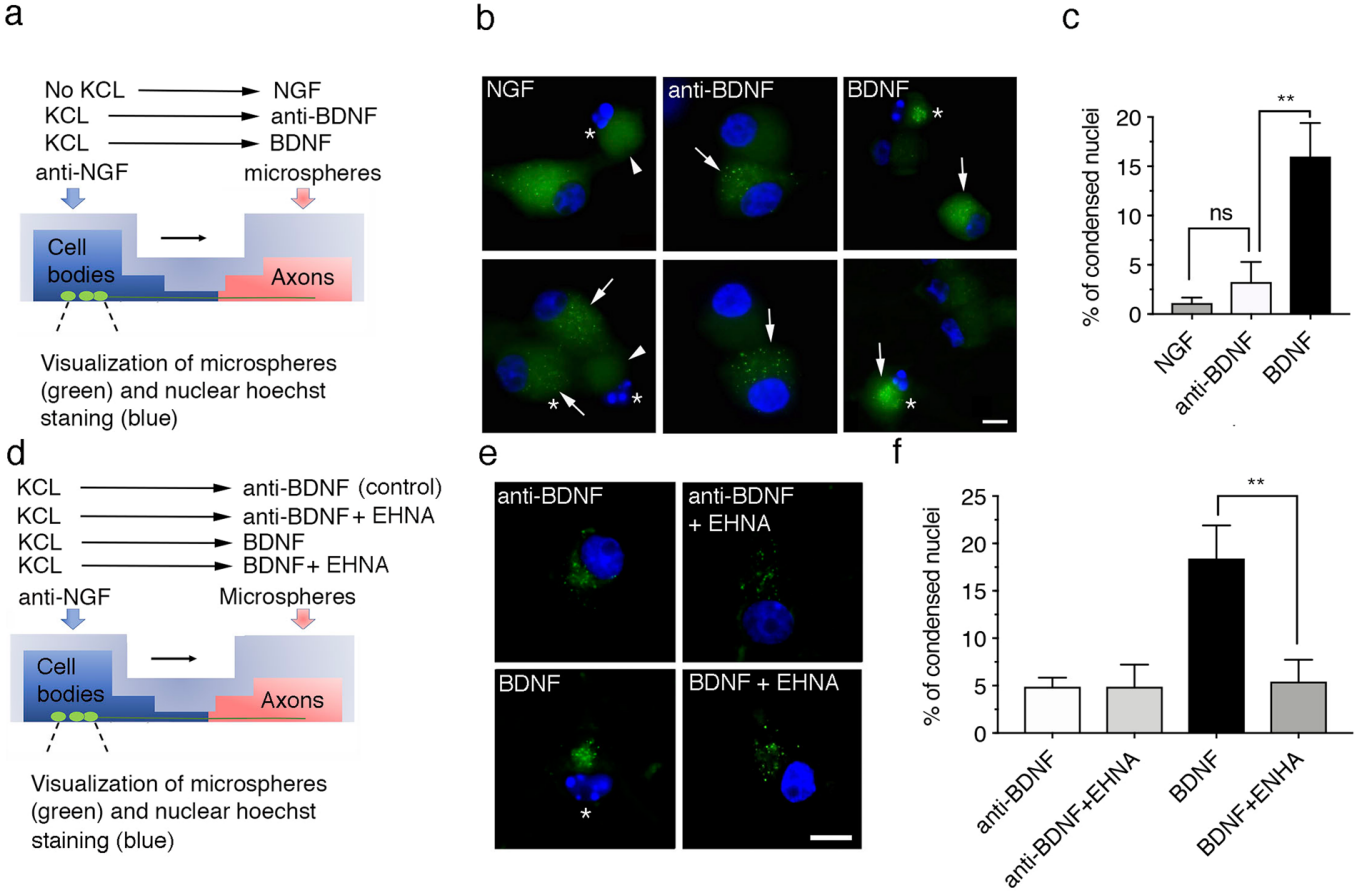
The addition of BDNF to the axons of sympathetic neurons induces retrograde cell death in a dynein-dependent manner. (**a**) Illustration of the design of the experiment used to study retrograde death signaling mediated by BDNF. (**b**) Visualization of the cell bodies of compartmentalized sympathetic neurons in which the axon compartment was treated with microspheres labeled with Alexa Fluor 488 (green) and NGF, 12.5 mM of KCl and an anti-BDNF antibody (No BDNF), or BDNF in the presence of 12.5 mM KCl (BDNF) for 30 hours. The addition of 12.5 mM of KCl prevented neuronal death in response to NGF withdrawal. The cell body compartment was treated with an anti-NGF antibody. Then, cultures were fixed and stained with Hoechst (blue) to visualize nuclei. Green fluorescence microspheres (microspheres labeled Alexa Fluor 488) were added to the axons of all cultures 24 hours before the start of the experiment to visualize the cell bodies of compartmentalized neurons. Only neurons that projected axons in the axon compartment are labeled with microsphere-Alexa Fluor 488, which are shown as bright green dots indicated by white arrows. Arrowheads indicate neurons with a faintly stained profile that do not contain green dots. These cells did not project axons into the axon compartment. Asterisks indicate apoptotic, condensed or fragmented nuclei. Cell bodies of noncompartmentalized neurons (microspheres-Alexa Fluor 488-negative), which were not affected by NGF in the axonal compartment, presented apoptotic nuclei, as expected. Similarly, neurons labeled with microspheres-Alexa Fluor 488 that were treated with BDNF in the axon compartment showed increased condensed or fragmented nuclei. Scale bar, 10 μm. (**c**) Percentage of apoptotic neurons observed after the different treatments. Fifty cells from 6–9 different compartmentalized chambers were quantified at each time point in three independent experiments. Statistical significance was analyzed using one-way ANOVA and Tukey´s multiple comparison test. **p < 0,009. (**d**) Illustration of the design of the experiment used to study the requirement for dynein in retrograde death signaling mediated by BDNF. (**e**) Visualization of neuronal cell bodies from compartmentalized sympathetic neuronal cultures treated as described in (d), as well as without (control) or with EHNA (dynein inhibitor). Scale bar, 10 μm. (**f**) Percentage of apoptotic neurons observed after the different treatments. Two hundred fifty cells from six different compartmentalized chambers were quantified at each time point in three independent experiments. Only cells that were retrogradely labeled with microspheres-Alexa Fluor 488 were subjected to the quantification of apoptosis. Statistical significance was analyzed using one-way ANOVA and Tukey´s multiple comparison test. **p < 0,0085.

We next investigated whether axonal p75 activation by BDNF is followed by neuronal degeneration, which is visualized as nuclear fragmentation, when the axons of CSCG are stimulated with BDNF. We treated the axonal compartment with fluorescently labeled microspheres that were retrogradely transported from axons to cell bodies to label neurons extending axons in the axonal compartment that were subsequently stimulated with BDNF (Fig. 2a,b). Consistently, when CSCG cultures were treated with NGF in the axonal compartment (and no KCL in the cell body compartment, see Fig. 2a) neurons labelled with fluorescent microspheres and therefore containing axons in the axonal compartment, possessed healthy nuclei. However, neurons that were not labelled with fluorescent microspheres and therefore survival signaling induced by TrkA/NGF were not triggered in the axon, showed nuclear fragmentation induced by NGF withdrawal (Fig. 2b,c), consistent with a previous report^30^.

Upon the addition of BDNF to the axonal chamber (in the presence of KCl in cell bodies; see Fig. 2d for a detailed description of the experimental design), axonally applied BDNF significantly increased nuclear fragmentation, indicating that BDNF binding to p75 induced retrograde killing (Fig. 2b,c). We tested whether the inhibition of dynein by EHNA reduced the BDNF-induced retrograde killing of neurons to determine whether the retrograde cell death induced by BDNF-mediated p75 activation in axons requires endosomal retrograde transport. As shown in Fig. 2e,f, blockade of the retrograde transport of p75 induced by dynein inhibition significantly reduced the apoptotic cell death induced by axonally applied BDNF. Based on our results, the application of BDNF to axons of CSCGs induces cell death, a process that required dynein-mediated transport of the death signal.

Of note, we treated the axons with BDNF for at least 16 hours to observe the accumulation of the retrogradely transported p75 protein in cell bodies. Consistent with the hypothesis that internalization and retrograde transport are required to induce neuronal death, neuronal cell death was observed after 30 hours of BDNF treatment. This time point is consistent with the kinetics of cell death observed in SCG mass cultures reported in the literature^8,23^.

### BDNF/p75-induced neuronal death requires Rab5 activity in cell bodies and axons

As shown in our previous studies, BDNF increases the clathrin-dependent endocytosis of p75 in noncompartmentalized SCGs^19,24,28^. After internalization, p75 was transiently associated with Rab5-positive endosomes but did not accumulate in this organelle and instead was sorted to recycling endosomes or CD63-positive multivesicular endosomes. When a constitutively active mutant of Rab5 was expressed in PC12 and SCG neurons, p75 was trapped in Rab5-positive organelles, indicating that p75 transits through Rab5-positive endosomes before being sorted to other endocytic organelles. Also, in motoneuron axons, p75 was internalized in Rab5-positive endosomes and sorted to Rab7 endosomes for retrograde transport^19,24,28^.

**Figure 3:**
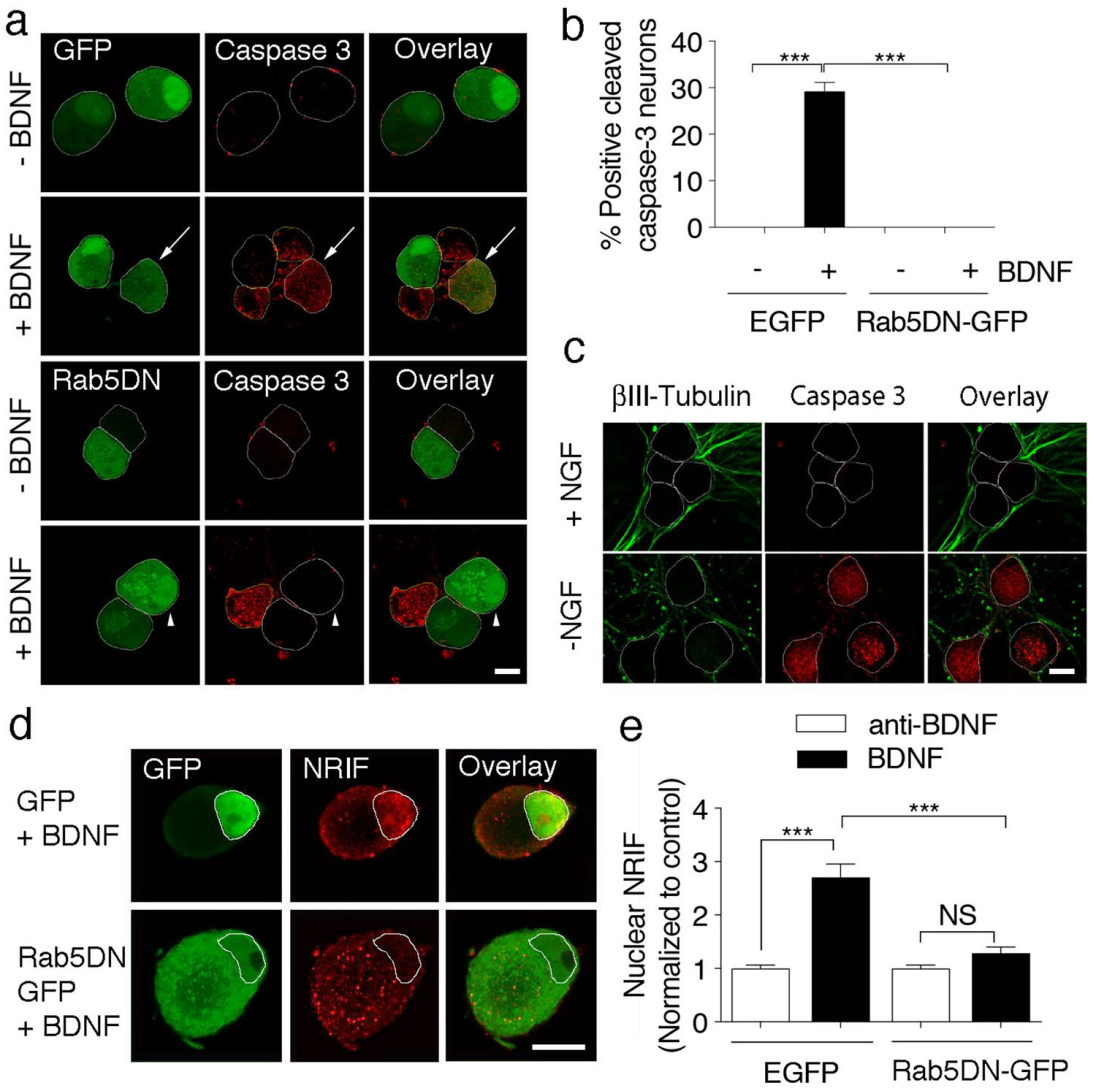
Rab5 activity is required for BDNF-induced cleavage of caspase 3 and NRIF nuclear accumulation in noncompartmentalized sympathetic neurons. (**a**) Confocal images of SCGs infected with a GFP or Rab5DN-GFP adenovirus (green) and immunostained for cleaved caspase 3 (red). At 6 DIV, neurons were infected with the GFP or Rab5DN-GFP adenovirus, and 24 h later, were treated with (+BDNF 150 ng/mL) or without BDNF (-BDNF) in the presence of 12.5 mM KCl and the anti-NGF (0,1 µg/mL) blocking antibody for 24 hours. Images show cytosolic cleaved caspase 3 accumulation induced by BDNF in GFP-positive and -negative cells, but not in Rab5DN-GFP-positive cells. Arrows indicate a GFP-positive neuron labeled with the cleaved caspase 3 antibody. Arrowheads indicate Rab5DN-GFP-positive neurons that were not stained with the cleaved caspase 3 antibody. Scale bar, 10 µm. (**b**) Quantification of cleaved caspase 3 staining in GFP- or Rab5DN-GFP-positive cells normalized to the total number of cells quantified. Only green cells infected with GFP or Rab5DN-GFP were considered. Two to three hundred neurons from 3 different experiments were quantified at each time point. Statistical significance was analyzed using two-way ANOVA and Tukey’s multiple comparison test. ****p < 0.0001. Scale bar, 10 µm. (**c**) Confocal images of SCGs grown for 6 DIV in mass cultures in the presence (+NGF, 50 ng/mL) or absence of NGF for 24 hours and immunostained with antibodies against βIII-tubulin (green) and cleaved caspase 3. As expected, NGF deprivation increases cleaved-caspase 3. Scale bar 10 µm. (**d**) Visualization of neuronal cell bodies in SCG cultures infected with the GFP or Rab5DN-GFP adenovirus (green), immunostained with antibodies against NRIF (red), and labeled with Hoechst to stain the nucleus (not shown, white circles label nuclear staining). Neurons were treated with (+BDNF, 150 ng/mL) or without BDNF for 30 hours in the presence of 12.5 mM KCl. NRIF accumulates in the nucleus of cells treated with BDNF and expressing GFP, but not in cells expressing Rab5DN-GFP. The right panel shows quantification of nuclear NRIF normalized to T0 hour. Scale bar, 10 µm. Thirty-six to 46 cells from 3 different experiments were quantified at each time point. Statistical significance was analyzed using two-way ANOVA and Tukey’s multiple comparison test. ***p < 0.0002. Scale bar, 10 µm.

Whether a Rab5-dependent sorting step is required for p75-mediated death signaling in the cell bodies or axons of SCG neuron cultures is unknown. To address this issue further, we first infected noncompartmentalized SCG cultures with an adenovirus driving the expression of GFP (control) or a dominant negative mutant of Rab5 (Rab5DN) and tested whether Rab5DN decreases markers of apoptosis, the activation of caspase 3 and the nuclear accumulation of NRIF. As shown in Fig. 3a,b, the reduction in Rab5 activity decreased the BDNF-induced cleavage of caspase 3. We used NGF withdrawal as a positive control for caspase 3 activation and observed a similar pattern of immunostaining compared to BDNF-induced apoptosis (Fig. 3c). In sympathetic neurons, p75 activation by BDNF in the soma has been shown to induce apoptotic cell death in an NRIF-dependent manner^8^. Therefore, we tested whether the BDNF-induced nuclear translocation of NRIF was reduced by the expression of RAB5DN. The expression of the Rab5 mutant significantly reduced BDNF-induced NRIF accumulation in the nucleus to the basal levels (Fig. 3d,e). We also analyzed whether the expression of Rab5DN reduced the endocytosis of p75 and found that the expression of the mutant did not affect ligand-dependent p75 endocytosis (Supplementary Figure 3).

Thus, p75-mediated neuronal death requires the transit of the receptor through a Rab5-positive endosome.

**Figure 4:**
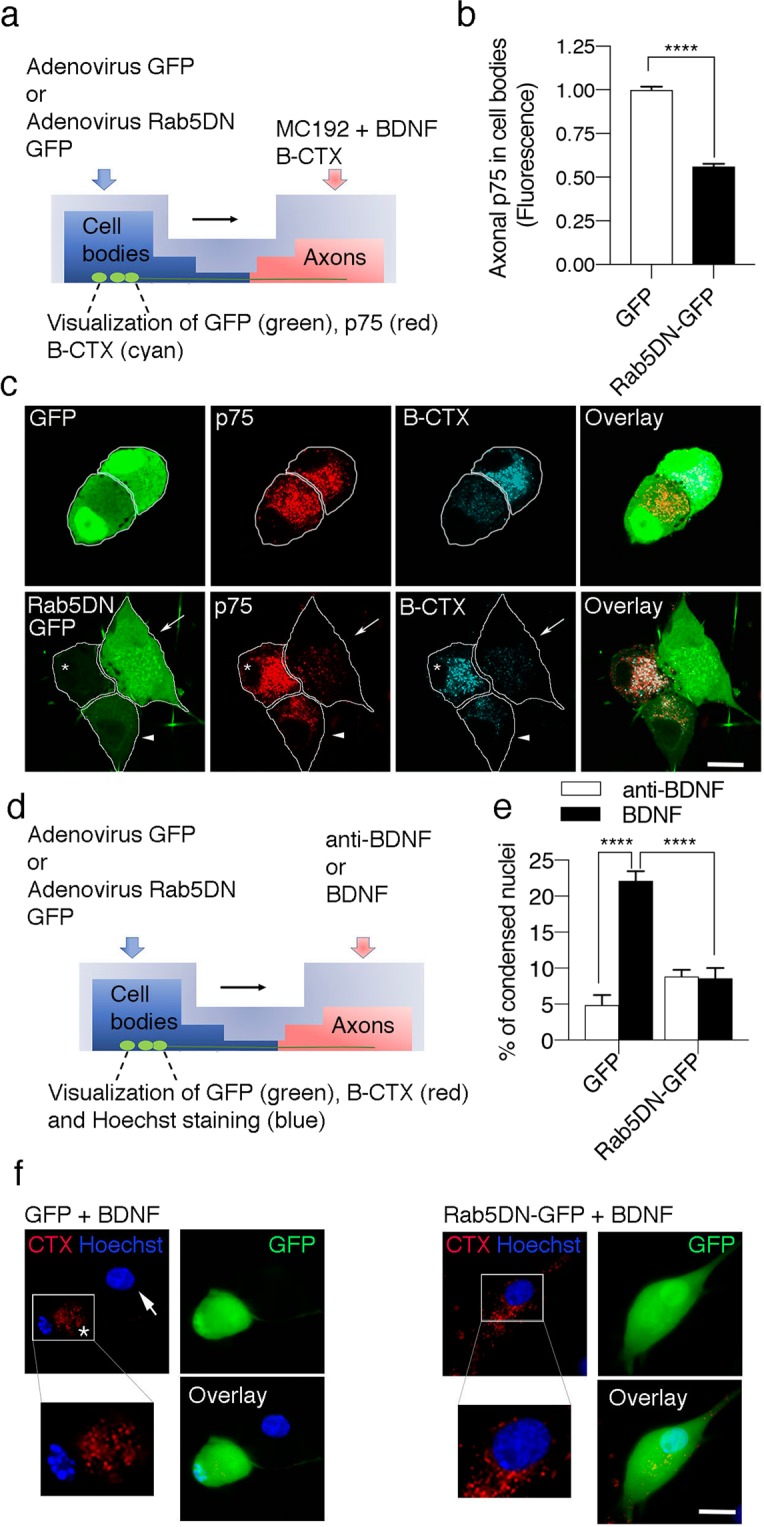
Rab5 activity is required for p75 retrograde transport and axonal BDNF-induced death signaling in sympathetic neurons. (**a**) Illustration of the design of the experiment used to study Rab5-dependent p75 retrograde transport and signaling in sympathetic neurons. Quantification (**b**) and visualization (**c**) of axonally labeled p75 in the cell bodies of compartmentalized sympathetic neurons. (**b**) Levels of axonally labeled p75 in the absence (control, GFP) or presence of a dominant negative Rab5 mutant (Rab5DN-GFP). Sixty cells from five different compartmentalized chambers were quantified. Statistically significant differences were analyzed using a two-tailed Mann-Whitney test. ****p < 0,0001. (**c**) After 5 days in culture, sympathetic neurons were transduced with the adenovirus driving the expression of GFP (green) or a dominant negative mutant of Rab5 fused to GFP (Rab5DN-GFP, green). Twenty hours after the infection, the axon compartment was treated with BDNF, MC192-Alexa Fluor 594 (to label p75, red) and the B subunit of the cholera toxin conjugated to Alexa Fluor 647 (B-CTX, cyan) for 16 hours at 37 °C to label compartmentalized neurons. Scale bar, 10 μm. Only cells that were retrogradely labeled with B-CTX-Alexa Fluor 647 were used to quantify the retrograde transport of p75. The white arrow indicates a Rab5DN-GFP labelled neuron with reduced levels of p75. The arrowhead indicates a neuron expressing lower labels of Rab5DN-GFP when compared to the neuron labelled with a white arrow. Consistently, this neuron shows increased levels of p75. The asterisk indicates a neuron that does not express Rab5DN-GFP. This neuron expresses greater levels of p75 compared to neurons labelled with Rab5DN-GFP. (**d**) Illustration of the design of the experiment used to study Rab5-dependent retrograde signaling of p75 and quantify axonal BDNF-induced death signaling in sympathetic neurons. Sixty cells from seven different compartmentalized chambers were quantified at each time point. Statistically significant differences were analyzed using two-way ANOVA and Tukey’s multiple comparison test. ****p < 0,0001. Only cells that were retrogradely labeled with B-CTX-Alexa Fluor 647 were used to quantify apoptosis. (**e-f**) Quantification (**e**) and visualization (**f**) of apoptosis of neuronal cell bodies from compartmentalized sympathetic neuronal cultures expressing GFP and Rab5DN-GFP (in green). Axons were treated with B-CTX-Alexa Fluor 647 (red) to label compartmentalized neurons. Condensed or fragmented nuclei were labeled with Hoechst (blue). The cell enclosed in a white square was magnified to enable the better visualization of the healthy or condensed nucleus labeled with an asterisk. The white arrow indicates a healthy nucleus that is not labeled with B-CTX-Alexa Fluor 647. Scale bar, 10 μm.

We infected the cultures with an adenovirus driving the expression of GFP or Rab5DN-GFP to further explore whether the retrograde transport of p75 and retrograde killing of CSCGs induced by axonal BDNF require Rab5 activity (Fig. 4a,d). CSCGs cultures were treated with fluorescently labelled B-subunit of cholera toxin (B-CTX) in the axonal compartment to retrogradely label neurons having axons in the axonal compartment. Just neurons labelled with B-CTX in the cell bodies where included in the analysis. The expression of Rab5DN significantly reduced the retrograde transport of p75 (Fig. 4b,c) and the retrograde cell death observed upon the axonal application of BDNF (Fig. 4e,f). Consistent with these findings, p75 was endocytosed and partially colocalized with endogenous Rab5-positive endosomes in axons and growth cones (Supplementary Figure 1).

Taken together, these results provide evidence that BDNF binds p75 on axons and induces the subsequent internalization and retrograde transport of the receptor to cell somas through a process that requires Rab5-mediated endosome sorting.

### BDNF-induced activation of JNK in axons increases the internalization and subsequent retrograde signaling of p75

**Figure 5:**
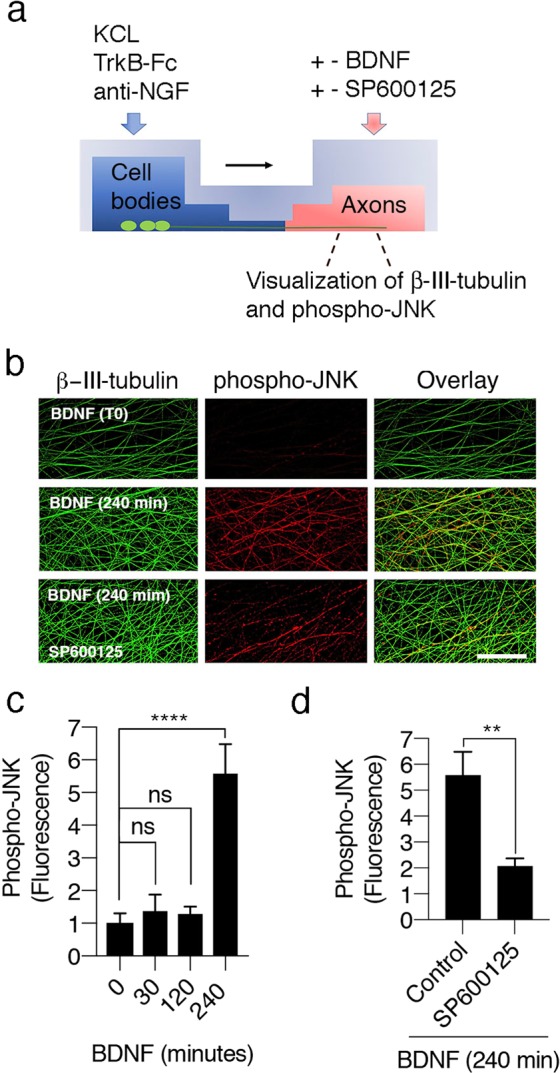
JNK is activated by BDNF applied to the distal axons of sympathetic neurons. (**a**) Illustration of the design of the experiment used to study BDNF-induced JNK activation in the axons of SCG neurons. (**b**) Visualization of distal axons from 7 DIV compartmentalized sympathetic neurons in which the axonal chamber was with BDNF (150 ng/mL) in the presence of 12.5 mM of KCl for 0 (T0), 30, 120 or 240 minutes. As indicated in (**a**), cell bodies were maintained in 12.5 mM of KCl and TrkB-Fc (150 ng/mL) without BDNF. Then, cultures were fixed and immunostained for phospho-JNK (red) and βIII-tubulin (green). Scale bar, 50 µm. (**c**) Quantification of axonal phospho-JNK levels after BDNF stimulation. Six to seven different compartmentalized chambers were quantified at each time point in three independent experiments. Values were normalized to T0. Statistical significance was determined using one–way ANOVA followed by Tukey’s multiple comparison test. ****p < 0.0001. (**d**) Quantification of axonal phospho-JNK levels after BDNF stimulation (240 min) in the absence (control) or presence of SP600125. Statistical significance was determined using two-tailed unpaired t-tests with Welch’s correction. **p < 0.0078.

Pathways leading to JNK activation are among the cell death signaling pathways activated by p75^5–7^. Therefore, we studied whether the addition of BDNF to the axons of sympathetic neurons induced JNK phosphorylation by performing immunofluorescence staining with a specific antibody against the phosphorylated form of activated JNK (Fig. 5a), validated in a previous study^31^. When we added BDNF to axons in microfluidic devices, JNK activity was significantly increased by BDNF in a time-dependent manner (Fig. 5b,c). The phospho-JNK staining was specific, since axons treated with BDNF and SP600125, an inhibitor of JNK, exhibited decreased axonal phospho-JNK staining (Fig. 5b,d). The kinetics of JNK activation resemble the kinetics of p75 internalization in SCGs neurons^24^. Upon the addition of BDNF, in a previous study using an antibody feeding assay we observed a delay of at least 60 minutes before p75 internalization was visualized, reaching a steady state after 4 hours of BDNF treatment in SCG neurons^24^. Consistent with these results, JNK was activated in axons 4 hours after the application of BDNF (Fig. 5b,c). We expected to visualize increased JNK activity after 2 hours of BDNF treatment; however, the immunofluorescence staining might not be sufficiently sensitive to distinguish the increase in JNK activity over basal levels at early time points.

**Figure 6:**
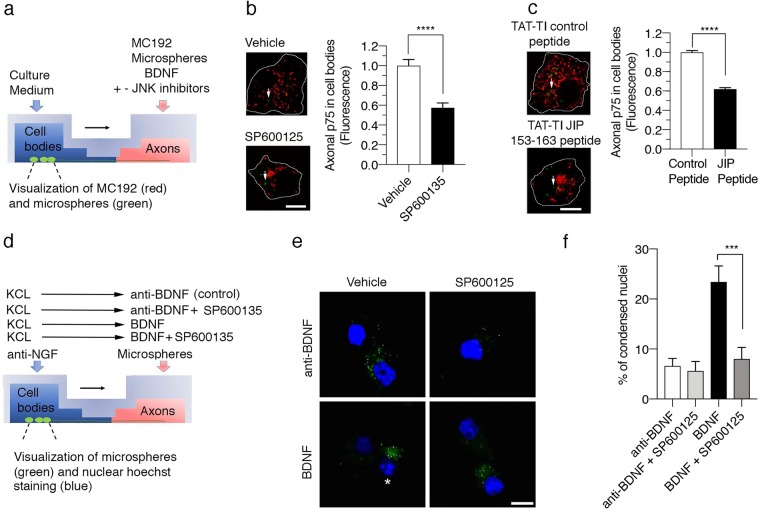
JNK activity is required for the retrograde transport and apoptotic signaling triggered by the application of BDNF to the axons of sympathetic neurons. (**a**) Illustration of the design of the experiment used to study the requirement for JNK activation in the retrograde transport of the p75 neurotrophin receptor in SCG neurons. (**b**) and (**c**) Visualization of cell bodies from 6 DIV compartmentalized sympathetic neuron cultures in which the axonal chamber was treated with microspheres-Alexa Fluor 488 (green) and 12.5 mM KCl in the presence of BDNF (200 ng/mL) for 16 hours, and then treated with the vehicles used to dissolve SP600125 or SP600125 (**b**) and or TAT-TI-control peptide or TAT-TI-JIP 153–163 (**c**). Right panels show the quantification of retrogradely transported p75 under control conditions or after treatment with the JNK inhibitors indicated in (**b**) and (**c**). White arrows indicate green microspheres. Data from 200 cells in 7 different compartmentalized chambers were averaged at each time point. Statistical significance was analyzed using a two-tailed Mann-Whitney test. ****p < 0,0001. (**d**) Illustration of the design of the experiment used to study the requirement for JNK activation in death signaling induced by p75 neurotrophin receptor in SCG neurons. (**e**) Visualization of neuronal cell bodies from compartmentalized cultures in which the axonal chamber was treated with BDNF and Alexa Fluor 488-labeled microspheres for 30 hours at 37 °C in the absence (control) or presence of the JNK inhibitor SP600125. Apoptosis was visualized by fragmented nuclei stained with Hoechst (blue), as indicated by white asterisks. Scale bar, 10 μm. (**f**) Percentage of apoptotic cells following the axonal BDNF treatment. Two hundred fifty cells from five different compartmentalized chambers were quantified at each time point. Statistical significance was determined using one-way ANOVA and Tukey’s multiple comparison test. ***p < 0,0002.

We noted possible correspondence for the delays in activating JNK and the accumulation of axonal p75 in cell bodies. Indeed, axons were treated with BDNF for at least 16 hours before observing accumulation of p75 in cell bodies (see below and Supplementary Figure 1), consistent with the hypothesis that delayed JNK activation delays the internalization and transport of p75. To test this hypothesis, we asked if JNK activity in axons was required for the retrograde transport and cell death mediated by p75 (Fig. 6a,d, respectively). Inhibition of axonal JNK activity was achieved by applying two different JNK inhibitors that both specifically inhibit JNK activation: a pharmacological compound (SP600135) and a plasma membrane-soluble peptide (TAT-TI-JIP153–163, a short sequence derived from the JNK adaptor JIP)^32,33^. Treatment with either JNK inhibitor reduced the transport of p75 induced by the application of BDNF on axons (Fig. 6b,c). Thus, consistent with the need for dynein based retrograde transport of axonal p75 to induce neuronal death (Figs 1 and 2), inhibition of axonal JNK with SP600135 also reduced apoptotic cell death induced by axonal BDNF (Fig. 6e,f).

JNK inhibition did not cause major axonal perturbations, as shown by βIII-tubulin immunostaining of axons treated with a JNK inhibitor and compared to axonal metabolic inhibition, which causes axonal beading (Supplementary Figure 5). In summary, JNK activity is required for retrograde transport and retrograde cell death induced by BDNF binding to p75 in axons.

**Figure 7:**
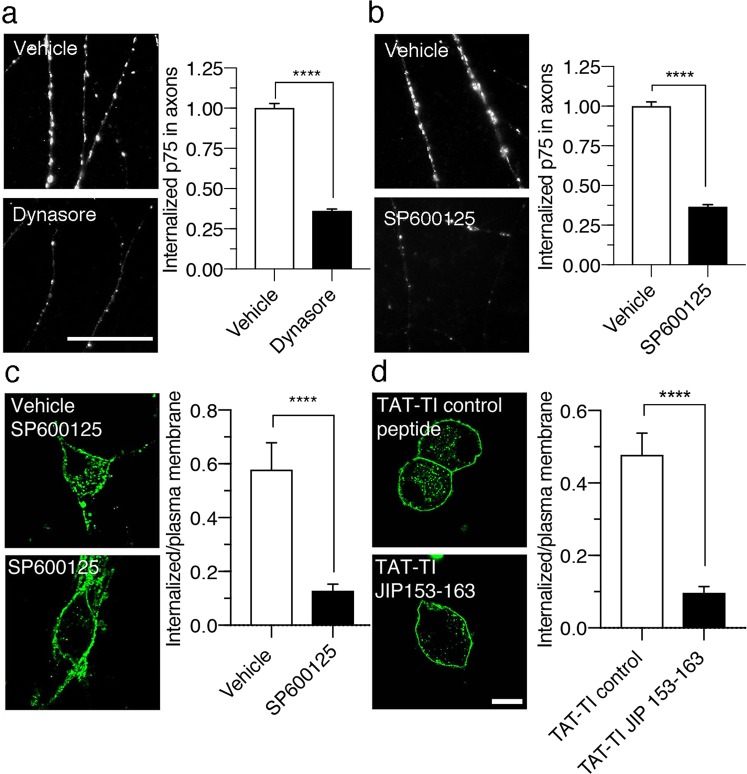
JNK promotes p75 internalization in the axons and cell bodies of sympathetic neurons. (**a**) and (**b**) Visualization of axons of compartmentalized cultures of sympathetic neurons treated with BDNF and MC192-pHRodo in the absence (vehicle) or presence of dynasore for 3–4 hours at 37 °C (**a**), or the absence (vehicle) or presence of SP600125 for 3–4 hours at 37 °C (**b**). The intensity of the pHRodo fluorophore is brighter at an acidic pH, indicating p75 internalization. An inhibitor of p75 internalization, dynasore, reduced the fluorescence associated with p75 internalization. Similar to the effect of dynasore, the presence of SP600125 reduces the fluorescence compared to vehicle conditions. Scale bar, 4,5 µm. Right panels show the quantification of the total fluorescence associated with 366 (vehicle for dynasore), 406 (dynasore), 268 (vehicle for SP600125), 227 (SP600125) axonal segments (9 μm long) from three independent compartmentalized neuronal cultures. Statistically significant differences were analyzed using a two-tailed Mann-Whitney test. ****p < 0,0001. (**c**) and (**d**) Confocal microscopy images of sympathetic neurons treated with BDNF (150 µg/mL) and MC192-Alexa Fluor 594 (3 μg/mL, green, to label p75) for 4 hours at 37 °C in the absence (vehicle and TAT-TI control peptide) or presence of the JNK inhibitors, SP600125 (10 µM) (**c**) or TAT-TI-JIP 153–163 peptide (1 μM) (**d**). Scale bar, 10 μm. Right panels show the levels of internalized p75 after different treatments (relative fluorescence normalized to cell surface p75). Sixty-five cells from three independent experiments were quantified. Statistically significant differences were analyzed using a two-tailed Mann-Whitney test. ****p < 0,0001.

The observation that the inhibition of retrograde JNK reduced the retrograde transport of p75 prompted us to study whether this process was caused by reduced axonal internalization or transport of the receptor. First, we evaluated p75 internalization using an MC192 monoclonal antibody labeled with the pH-sensitive dye pHRodo, which exhibits a low fluorescence intensity at a neutral pH and fluoresces brightly at acidic pH values, i.e., at ~6.5 or lower values (see the Life Technologies data sheet), to assess the role of JNK in mediating p75 internalization in axons. When CSCG axons were incubated with MC192-pHRodo in the presence of BDNF, we observed an increase in axonal fluorescence (Fig. 7a). This increase was abolished when axons were treated with dynasore (Fig. 7a), a drug known to reduce the dynamin-dependent internalization of p75 and other receptors^2,34^. Similarly, the treatment of axons with JNK inhibitor SP600125 reduced the fluorescence intensity, indicating that JNK activation increased the internalization of p75 in axons (Fig. 7b). We also studied whether JNK inhibition reduced the internalization of p75 in the cell body. Upon JNK inhibition by SP600125 (Fig. 7c) or TAT-TI-JIP153–163 (Fig. 7d), ligand-dependent p75 internalization was substantially reduced in neuronal cell bodies (Fig. 7c,d). The decrease in p75 internalization was not due to a general inhibition of receptor internalization because the JNK inhibitors did not reduce the internalization of transferrin (Supplementary Figure 6). The reduction in p75 internalization was confirmed with electron microscopy, where, in the presence of SP600125, the QDot-labeling p75 were clearly located outside the cell (Supplementary Figure 7). These data are evidence that internalization of p75 in the cell bodies and axons of sympathetic neurons requires JNK activity.

**Figure 8:**
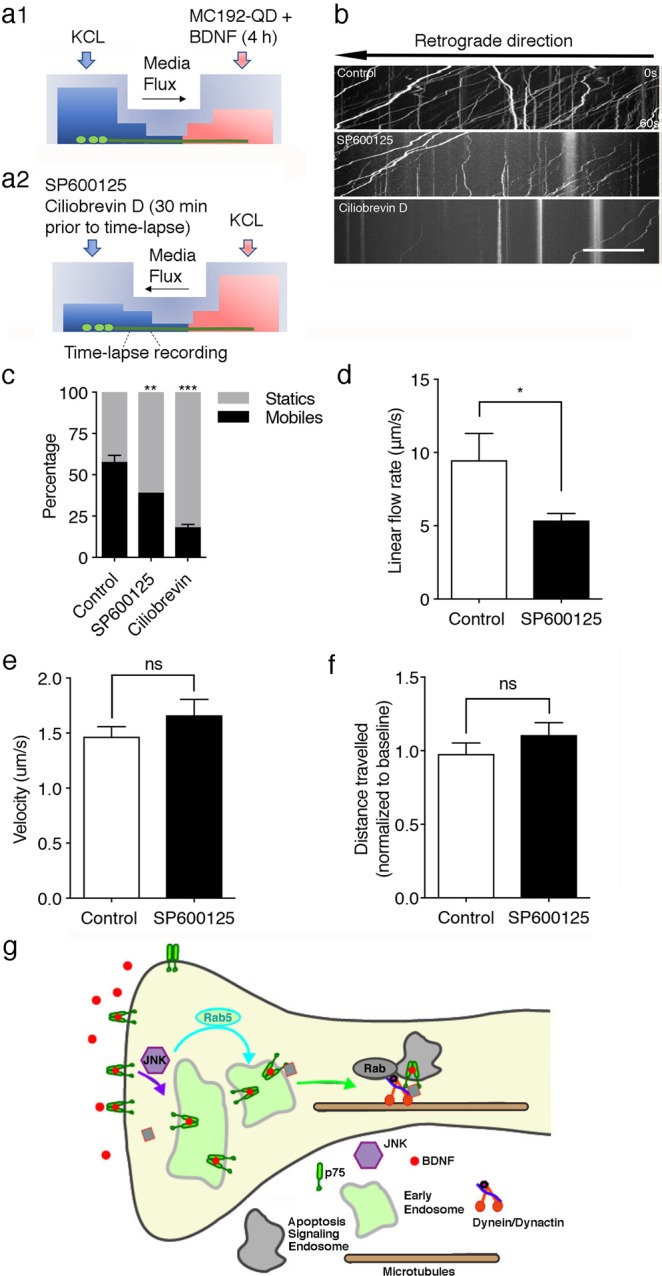
JNK is required for the efficient retrograde transport of p75-positive endosomes. (**a**) Schematic of the methodology; the axonal compartment of sympathetic neurons was treated with MC192 conjugated with QD605 and BDNF for 4 hours. Thirty minutes prior to the time-lapse recording, the medium was changed in both compartments to remove the MC192-QDot. Then, the cell body compartment was treated with SP600125 or ciliobrevin D (20 μM) for 30 minutes. Finally, time-lapse images of the cell body compartment were captured at a site proximal to the microgrooves to evaluate the local effects of the inhibitors on the axonal transport of previously endocytosed MC192-QDot. (**b**) Representative kymograph of the retrograde transport of MC192-QDot in axons treated with vehicle, SP600125 or ciliobrevin D. Scale bar, 20 μm. (**c**) Quantification of the number of mobile (>5 μm traveled) and static (<5 μm traveled) particles in axons. Twelve videos from 4 chambers per condition in 2 independent experiments were quantified and analyzed using two-way ANOVA and Tukey’s multiple comparison test. **p < 0.005 and ***p < 0.001. (**d**) Quantification of endosomal flux, as evidenced by the number of dots observed during 30 seconds in 50 μm of axon multiplied by the average velocity of the particles during the time-lapse in each video. Three videos per chamber under each condition were quantified in 2 independent experiments. Student’s t-test: *p < 0.05. (**e**) Graph of the average velocity of the particles under each condition. Fifty-eight to 106 particles per condition were quantified in 2 independent experiment. Statistical significance was determined using Student’s t-test. p = 0.5868. (**f**) Graph indicating the ratio of the average distance traveled by the moving particles under each condition. Fifty-eight to 106 particles per condition were quantified in 2 independent experiments. Statistical significance was determined using Student’s t-test. p = 0.3249. (**g**) In sympathetic neurons axons, BDNF bound to p75 is internalized in a JNK-dependent manner to Rab5 positive endosomes and then is sorted to apoptosis-signaling endosomes for dynein mediated retrograde transport.

To study whether JNK is also required for p75 endosomal transport following BDNF treatment, we performed live-cell imaging experiments of axonally endocytosed p75 in the cell body compartments in the absence or presence of dynein or JNK inhibitors. To carry out these studies, we first treated the axonal compartment with MC192-QD and BDNF for four hours to promote p75 internalization and transport under conditions that prevented extracellular BDNF and MC192-QD from accessing cell bodies, as illustrated in Fig. 8a1. Then, the medium in the axon compartment was replaced with fresh media lacking BDNF and MC192-QD; at the same time the medium and the cell body compartment was treated with the dynein inhibitor ciliobrevin D^35^ or SP600125 for 30 minutes. During this phase media flux prevented movement of ciliobrevin or S0600125 from the cell body to the axon compartment. Then, time-lapse imaging in the proximal axons present in the cell body compartment recorded movement of MC192-QD, marking endocytosed p75, to compare the control and inhibitors, as shown in Fig. 8a2. Control cultures showed several p75-positive endosomes arriving in proximal axons and cells bodies, as documented in the kymograph of the cell body compartment (Fig. 8b). Inhibition of dynein halted almost all transport arriving in the cell body compartment (Fig. 8c). JNK inhibition also significantly increased the number of static vesicles (Fig. 8c) thus reducing the flux of p75-containing endosomes (Fig. 8d) without changing the velocity or the distance travelled by the mobile vesicles (Fig. 8e,f). Thus, the activation of p75 in axons increases JNK activity, a process that is required for the internalization of p75 to Rab5-positive signaling endosomes. Additionally, JNK activity modulates dynein-dependent retrograde transport of p75, thus facilitating the propagation of p75 signaling to effect activation of apoptosis in cell bodies (Fig. 8g).

## Discussion

Based on our findings, the binding of BDNF to p75 in the axons of sympathetic neurons increases the internalization and transport of the full-length p75 receptor to induce cell death in a dynein- and Rab5-dependent manner. Additionally, the activation of p75 in axons increases axonal JNK activity that is required for p75 internalization and transport and subsequent neuronal degeneration. Our results add to the evidence indicating that the activation of p75 in axons induces retrograde killing^16,17^, and we are the first group to report the requirements for JNK activation and Rab5-mediated endocytic sorting followed by dynein-mediated transport in this process (Fig. 8g). Our results are consistent with a recent report showing that BDNF-induced retrograde killing requires the internalization of axonal p75^16^.

Importantly, the p75-dependent induced death of SCG neurons is a complex process requiring long-lasting activation of JNK and NRIF nuclear accumulation^8,23^. JNK activation is also necessary for the proteolytic processing of full-length p75 (FL-p75) into a carboxy-terminal fragment (CTF-p75) and a soluble intracellular domain (ICD-p75). This process is required for NRIF nuclear accumulation and subsequent neuronal death^23^. According to a recent study by Pathak and colleagues, axonal p75 processing is necessary for HDAC1-mediated deacetylation of the dynactin subunit p150^Glued^, a process that enhances the interaction of dynactin with dynein and is required for BDNF-dependent retrograde killing of SCGs^16^. In this study, however, only a small proportion of axonal p75 was processed to ICD-p75.

Consistent with these findings, in our study, the extracellular domain (labeling the FL-p75) is present during retrograde transport of p75-containing endosomes (since the label used to track p75 binds to the extracellular domain of the receptor) and shares the dependence on JNK activation and the activities of both Rab5 and dynein with the death signaling endosomal compartment (Figs 2, 4 and 6). Intriguingly, for BDNF-mediated retrograde killing of SCGs, p75 cleavage is only required in the axonal compartment and not in the cell bodies^16^. This observation prompts question of whether retrograde axonal killing induced by BDNF requires the same mediators as when BDNF induces death by application to cell bodies. When cell death is triggered by stimulating p75 receptors in cell bodies of SCGs, p75 processing and nuclear NRIF accumulation are required^8^. Alternatively, ICD-p75 may be required for HDAC1-induced deacetylation of dynactin subunit p150^Glued^ to increase the retrograde transport of p75-FL in a process that requires Rab5-dependent sorting to signaling endosomes.

Our results showing a reduction in NRIF levels in the nucleus in noncompartmentalized SCG cells treated with BDNF and expressing a dominant negative Rab5 are consistent with the hypothesis that internalization and the transit through Rab5-positive endosomes are required for p75 processing and further transport in cell bodies and axons. As shown in our previous study, p75 is processed in the endocytic pathway in PC12 cells^36^. Since CTF-p75 has been observed in the axonal endosomes of motor neurons under pathological conditions^18^, p75 may be cleaved during transit, and the ICD-p75 fragment could be sequestered and protected from the proteasome in CD63-positive multivesicular endosomes^16,24^. Additional studies are required to explore the locus of p75 processing and define the events catalyzed by death-signaling endosomes containing p75.

An interesting question is how JNK activity facilitates the internalization of p75. We are not aware of data indicating that JNK substrates regulate receptor internalization or that JNK phosphorylation of membrane receptors is involved in this process. One possibility is that p75 interacts with the adaptor JIP3 (JNK-interacting protein 3) to increase its association with JNK or that p75 is directly phosphorylated by JNK3 to promote its internalization (see below). The requirement of JNK activity for p75 transport (Fig. 8) is also intriguing and deserves further investigation. We postulate that an unbiased approach to studying the phosphorylation of JNK substrates and interacting partners after p75 activation might help unravel this mechanism.

Early work on p75 and TrkA signaling has suggested that p75 and TrkA are counterpoised in determining biological outcomes. For example, p75-induced JNK activation is reduced in oligodendrocytes upon the exogenous expression and activation of components of the Trk signaling pathway^12^. Similarly, elegant genetic experiments published by Miller and colleagues have shown that this mechanism is also employed during the development of SCGs; p75 cell death signaling depends on JNK, and the expression of the p53 transcription factor is downregulated by NGF-induced TrkA activation in SCG axons^37^. Based on the results from our experiments, the activation of axonal p75 contributes to the competition between life and death decisions made in developing cell bodies of SCG neurons. This process might also be activated during neurodegeneration because JNK has been shown to play a role in signaling in response to axonal injury and degeneration^38^, and p75 is upregulated upon injury in subjects with neurodegenerative diseases^4^. Supporting this hypothesis, we report increased JNK activity upon the binding of BDNF to p75 in axons (Fig. 5); increased expression of p75 in the context of neurodegeneration would contribute to death signaling upon stress-induced JNK activation. Interestingly, when JNK is activated by neurotrophin withdrawal in the axons of sensory neurons via DLK kinase, this process results in c-Jun phosphorylation and apoptosis after the redistribution of JNK to the cell body, a process that requires retrograde transport mediated by dynein. In addition, JNK3-positive vesicles have been reported in axonal injury responses through an interaction with dynein via JIP3 (a.k.a. Sunday driver) as an adaptor for retrograde transport^31,38–40^. Therefore, future studies examining a role for JNK in p75 retrograde signaling in the context of both the developing and degenerating nervous system are warranted.

While confirming a role for Rab5, our data do not identify the compartment in which transport occurs. In the soma of SCG neurons, p75 trafficking is complex, and we have shown that upon BDNF addition, internalized p75 is trafficked from Rab5- to Rab11-positive endosomes and then to CD63-positive multivesicular bodies (MVB)^24^. These MVBs are not positive for Rab7 and are specialized for exosomal release. On the other hand, in motor neurons, p75 has been shown to transiently interact with Rab5 before sorting to Rab7-positive endosomes, where retrograde transport of p75 occurs^19^. Notably, Rab7/p75-positive endosomes are also positive for TrkB and a fragment of the tetanus toxin^19^. As shown in our previous studies using PC12 cells, which express both p75 and TrkA, the addition of NGF results in the formation of distinct population of endosomes, some containing both p75 and TrkA and others containing just one receptor. The different types of endosomes might result in part from different rates of internalization of TrkA and p75^24,28^. Therefore, organelles containing both p75 and Trks may be different than organelles that only contain p75. Indeed, Rab7-positive MVBs were recently shown to be more abundant carriers of axonal NGF-TrkA-positive signaling endosomes in sympathetic neurons^41^. Additionally, ICD-p75 localizes to CD63-positive organelles in axons^16^.

In conclusion, the binding of BDNF to p75 on axons in developing SCG neurons induces JNK activation followed by neuronal cell death. The process requires endocytosis and retrograde endosomal transport of p75 in a Rab5- and dynein-dependent manner. Our findings are also consistent with a defining role for JNK activation in axons in regulating the endocytosis and trafficking of BDNF/p75-containing endosomes that signal cell death. Our studies open exciting new areas of inquiry regarding the creation, composition and regulation of death signaling endosomes and their roles in both the developing and degenerating nervous system.

## Methods

All experiments were conducted in accordance with approved guidelines of CONICYT (Chilean National Commission for Scientific and Technological Research). Experiments with vertebrates were approved by the bioethical committee of the Faculty of Biological Sciences at the Catholic University of Chile.

### Materials

NGF, BDNF, anti-BDNF and the monoclonal MC192 anti-p75 antibody were purchased from Alomone Labs (Jerusalem, Israel). Bovine pancreatic trypsin, SP600125, TAT-TI-JIP 153–163 and the TAT-TI control peptides were purchased from Calbiochem-Merck (Darmstadt, Germany). DNAse I, D-lysine, laminin, dynasore, EHNA (erythro-9-amino-ß-hexyl-α-methyl-9H-purine-9-ethanol hydrochloride), aphidicolin, the anti-βIII-tubulin antibody and Mowiol 4–88 were purchased from Sigma (St. Louis, MO, USA). The blocking anti-BDNF antibody was purchased from Promega (Madison, WI, USA), and the blocking anti-NGF antibody was purchased from Chemicon (California, USA). Transferrin-Alexa Fluor 594, anti-mouse-Alexa Fluor 488 and 555, anti-rabbit-Alexa Fluor 488 and 647, anti-goat-Alexa Fluor 488, Hoechst 33258, Alexa Fluor 488-labeled microspheres (40 nm), Alexa Fluor 488-5TFP, Alexa Fluor 594-conjugated carboxylic acid succinimidyl ester, pHRodo, streptavidin-QDots 655, glutamine, penicillin and streptomycin were purchased from Molecular Probes (Eugene, OR, USA). Protease-free bovine serum albumin (BSA) was purchased from Jackson ImmunoResearch (West Grove, PA, USA). The Map1B antibody and IgY chicken serum were purchased from Santa Cruz Biotechnology (Santa Cruz, CA, USA). The Map2 antibody was purchased from Upstate-Millipore (Billerica, MA). The Rab5 antibody was purchased from Abcam. The phospho-JNK rabbit antibody was purchased from Cell Signaling Technology (Danvers, MA, USA). The anti-NRIF antibody has been described previously^23^ and was a gift from Dr. Bruce Carter at Vanderbilt University. The cleaved caspase-3 (Asp175) rabbit mAb and the phospho-JNK rabbit antibody was purchased from Cell Signaling Technology (Danvers, MA, USA). Gelatin from cold water fish skin was obtained from Sigma (St. Louis, MO). QD605-streptavidin conjugates were obtained from Invitrogen (Thermo Fisher Inc.) and Ciliobrevin D was purchased from Calbiochem-Merck (Darmstadt, Germany). The ultraculture medium was purchased from Lonza, and fetal bovine serum was purchased from HyClone (Thermo Fisher Scientific Inc.). EZ-link biotin-PEO-amine was purchased from Pierce (Rockford, IL, USA). The protein phosphatase inhibitors were obtained from Thermo Fisher Scientific Inc. TrkB-Fc and TrkA-Fc chimeric proteins were purchased from R&D Systems.

### Methods

#### Cell culture

Sympathetic neurons were dissected from the superior cervical ganglia (SCGs) of female and male Sprague-Dawley rats on postnatal day one. Ganglia were enzymatically dissociated in 0.5 mg/mL bovine pancreatic trypsin for 30 minutes at 37 °C and 0.2 mg/mL DNAse I for 5 minutes at 37 °C and finally mechanically dissociated. Neurons were plated on coverslips coated with poly-D-lysine and collagen when not cultured in compartmentalized chambers. SCGs were cultured with ultraculture medium supplemented with 6% fetal calf serum, 2 mM glutamine, 100 U/mL penicillin, 100 µg/mL streptomycin and 50 ng/mL NGF (SCG culture medium). The medium was supplemented with 3.3 μg/mL aphidicolin and 20 μM fluorodeoxyuridine to eliminate nonneuronal cells. Microfluidic chambers were produced as described by Park *et al*.^11^ and affixed to coverslips coated with 50 µg/mL poly-L-ornithine and 10 µg/mL laminin. Dissected neurons were seeded in the cell body compartment along with 50 ng/mL NGF, and medium containing 150 ng/mL NGF was added to the axon compartment to promote the projection of axons to this chamber. The neurons were grown for 5 to 7 days *in vitro* (DIV) to allow axons to project into the distal axon compartment. The difference in volume between the two compartments was maintained at 100 µL to ensure fluidic isolation during experiments.

#### Adenoviral production and amplification

The adenovirus driving the expression of EGFP was kindly donated by David Kaplan (Sick Children’s Hospital, Toronto, Canada). The Clontech plasmid containing the sequence of EGFP fused to the dominant negative form of Rab5 (Rab5DN-GFP) was kindly donated by Victor Faundez (Emory University, USA). The adenovirus containing the Rab5DN-GFP cDNA was prepared using a previously described (Luo *et al*., 2007; He *et al*., 1998) method for nonreplicative adenovirus production and generated using the AdEasy System (AdEasy™ Adenoviral Vector System). First, the Rab5DN-GFP sequence was subcloned into the shuttle vector (pShuttle-CMV, Catalogue #240081) and then, the shuttle vector was linearized with the Pme I enzyme and electroporated into BJ5183 bacteria (BJ5183-AD-1 Electroporation Competent Cells #200157-11 Stratagene) carrying the pAdEasy-1 plasmid that encodes the Adenovirus-5 genome (E1/E3 deleted). The final plasmid vector was produced by a double-recombination event between the cotransformed adenoviral backbone plasmid vector pAdEasy-1 and a shuttle vector carrying the gene of interest, thus generating a recombinant adenovirus genome that contains the gene of interest.

A biosafety level II chamber culture was used for adenovirus manipulation and infections. The recombinant adenoviral plasmids generated were then selected on kanamycin plates and confirmed by restriction digest, PCR and sequencing. The recombinant adenoviral plasmid was linearized by digestion with the Pac I enzyme and transfected into HEK293 cells. Viral particles were amplified to increase the viral titer in HEK293 cells. Finally, after several rounds of infections to increase the viral titer, the viral particles were purified and quantified using a viral particle assay (VIRAPUR TM, Adeno MINI Purification ViraKit™ 24-Use, Cat# 003059). Then, these viruses were used to infect SCGs.

#### Real-time microscopy

To study the retrograde transport of p75 in CSCGs, neurons were serum starved for 1 hour with DMEM supplemented with 25 mM HEPES without phenol red and 1 mg/mL BSA (incubation medium), and then the axons of SCGs were incubated with 12 μg/mL MC192-QDots and 200 ng/mL BDNF in incubation medium for 3 hours at 37 °C. Afterwards, axons were washed with incubation medium and 200 ng/mL BDNF were added to the axonal compartment and movies were taken using an inverted microscope Leica CTR6000 connected to a Rolera-MGI camera and to a system that controlled the temperature and CO_2_. Kymographs were obtained using the ImageJ program.

#### Retrograde transport of p75

Compartmentalized SCG neuronal cultures were incubated with fluorescence microspheres (labeled with Alexa Fluor 488) 24 hours before the addition of BDNF. This procedure allowed us to recognize the cell bodies projecting axons into the axonal compartment. Cultures were serum starved with incubation medium for SCGs supplemented with 12.5 mM KCl and 0.1 µg/mL anti-NGF for 1 hour to measure the retrograde transport of p75 in the cell bodies. Then, the axons were incubated with Alexa Fluor 594-conjugated MC192 (prepared using standard methods with Alexa Fluor 594-labeled carboxylic acid succinimidyl ester according to the manufacturer’s protocol), 200 ng/mL BDNF and green fluorescence microspheres in SCG incubation medium for 16 hours at 37 °C. Fluorescent microspheres were maintained in the cells throughout the experiment. The axonal compartment was treated with 1 μM EHNA and 10 μM SP600125 to inhibit dynein and JNK, respectively. The axonal chamber contained less volume than the cell body chamber in all steps (100 μl difference). In the experiments without BDNF, the axons were incubated with the anti-BDNF antibody in the axonal compartment. SCGs were fixed with 4% paraformaldehyde for 30 minutes at room temperature, washed with PBS and mounted in Mowiol mounting media. The fluorescence associated with the SCG cell bodies was quantified with the ImageJ program, and only cell bodies that were also labeled with fluorescence microspheres were analyzed. All the drugs were applied during the starving procedure, as well as during immunoendocytosis.

The retrograde tracer used in neurons infected with the adenovirus driving the expression of the dominant negative Rab5 mutant (Rab5DN) fused to EGFP (Rab5DN-GFP) or EGFP alone was the B subunit of the cholera toxin conjugated to Alexa Fluor 647 (B-CTX-Alexa Fluor 647) at a concentration of 1.3 μg/mL. B-CTX was applied 24 hours before the retrograde assay and maintained in the media throughout the experiment. Cells were infected 20 hours prior to the retrograde transport assay.

#### Retrograde neuronal cell death assay

The axonal compartment was incubated with fluorescence microspheres (labeled with Alexa Fluor 488) or B-CTX-Alexa Fluor 647 for 16 hours before the addition of BDNF to axons to study whether the application of BDNF to the axons of CSCG neurons (5-7 DIV) induced neuronal cell death. Then, the cultures were treated with incubation media supplemented with 12.5 mM KCl and 0.1 μg/mL of anti-NGF. In addition, the axonal compartment was incubated with 200 ng/mL BDNF for 30 hours. The effects of NGF withdrawal from neuronal cell bodies and rescue by NGF applied to axons were assessed by treating the neuronal cell body compartment with incubation media and the axon compartment with incubation media supplemented with 100 ng/mL NGF for 30 hours. Neurons that did not extend axons in the axon compartment were not rescued from NGF withdrawal-induced neuronal cell death. Chambers were washed, fixed with 4% paraformaldehyde, and the nuclei of cells in the cell body chamber were stained with Hoechst 33258. In experiments assessing the effects of dynein or JNK inhibitors, inhibitors were added together with the fluorescent retrograde marker and maintained during the BDNF treatment. The axonal compartment was treated with 1 μM EHNA and 10 μM SP600125 to inhibit dynein and JNK, respectively. Neurons were treated as described above in the section describing the inhibition of the retrograde transport of p75 by the expression of Rab5DN-GFP, but axons were treated with BDNF for 30 hours to assess the effect of Rab5 inhibition on apoptosis induced by retrograde BDNF signaling. A lower percentage of cells died in the present study than in mass cultures or compartmentalized cultures. A few technical aspects must be considered. First, we were unable to treat axons with BDNF for 48 hours^16^ because the neuronal death started to increase in the control cultures. Second, we only quantified cells that were double stained with retrograde tracers and Hoechst nuclear staining. Finally, we did not perform additional washes before adding BDNF; therefore, the maximum apoptosis was unlikely to be observed due to residual NGF activity. A neuron was scored as apoptotic if it contained a condensed or fragmented nucleus when stained with Hoechst and was costained with fluorescence microspheres or B-CTX-Alexa Fluor 647. See Supplementary Figure 1.

#### Immunoendocytosis of p75 in SCG cell bodies and axons

Cultures of SCG neurons (5–7 DIV) were serum starved with incubation medium (DMEM supplemented with 25 mM HEPES and 1 mg/mL BSA) for 1 hour at 37 °C to determine the effects of JNK inhibitors on the internalization of p75 in cell bodies. Then, SCG neurons were incubated with MC192-Alexa Fluor 594 (3 µg/mL) in incubation medium supplemented with 150 ng/mL BDNF or 60 µg/mL Transferrin-Alexa Fluor 594 at 37 °C for different times. Neurons were fixed with 4% paraformaldehyde for 15 minutes at room temperature. The cell surface-associated p75 or Transferrin was obtained by incubating SCG neurons with MC192-Alexa Fluor 594 or Transferrin-Alexa Fluor 594, respectively, at 4 °C for 2 hours (referred to as 0 minutes of internalization). JNK inhibitors were added to the incubation medium during the period of starvation and the immunoendocytosis procedure. SP600125 was added at a final concentration of 10 μM and the TAT-TI-JIP 153–163 peptides and the TAT-TI control peptide were used at a 1 μM final concentration.

Compartmentalized cultures of SCGs were serum starved with incubation medium for 1 hour at 37 °C to determine the effects of JNK inhibitors on the internalization of p75 in the axons of SCG neurons. Axons were incubated with MC192-pHRodo (10 μg/mL) in incubation medium supplemented with 150 ng/mL BDNF at 37 °C for 3–4 hours. JNK inhibitors were applied at the same concentration mentioned above, and dynasore was used as described in Escudero *et al*. 2014^24^. Neuronal cultures were not fixed, and photographs of axonal p75 were captured with an inverted microscope. The medium included the anti-BDNF antibody (10 μg/mL) in the cell body compartment to avoid p75 activation by endogenous BDNF during immunoendocytosis assays.

#### Immunofluorescence staining of compartmentalized cultures

Immunofluorescence staining of compartmentalized cultures was performed with a microfluidic chamber placed on a coverslip. Samples were fixed with 4% paraformaldehyde for 20 minutes at room temperature and then blocked and permeabilized with 0.1% Triton and 3% BSA in PBS for 3 hours at room temperature (IF incubation medium). All primary and secondary antibodies were diluted in IF incubation medium, and the samples were incubated overnight at 4 °C. The concentration of the anti-Map1B antibody was 0.7 µg/mL, and the dilutions of the anti-Map2, anti-BDNF and the secondary antibodies were 1:200, 1:200 and 1:400, respectively.

To visualized JNK activation by immunofluorescence, compartmentalized cultures were first serum starved for 1 hour in incubation media supplemented with 12.5 mM KCl and treated for different times (0–240 minutes) with BDNF (150 ng/mL) in incubation media supplemented with 12.5 mM KCl in the axon compartment and incubation media supplemented with 12.5 mM KCl and TrkB FC (200 ng/mL) to scavenge any secreted BDNF in the cell body compartment at 37 °C. After the treatment, cultures were fixed with 4% paraformaldehyde and permeabilized with 0.2% Triton and 5% fish gelatin in PBS supplemented with protein phosphatase inhibitors (PBS-PPI) for 1 hour at room temperature. Then, the axonal compartment was incubated with the phospho-JNK rabbit antibody (1:200) and anti-βIII-tubulin mouse antibody (1:1000) in PBS-PPI overnight at 4 °C. After several washes with cold PBS-PPI, the fixed cultures were incubated with donkey anti-rabbit and mouse secondary antibodies conjugated to Alexa Fluor 555 and Alexa Fluor 488 (1:300), respectively, in PBS-PPI for 1 hour at room temperature, washed (with PBS-PPI) and mounted in Mowiol.

#### Fluorescence microscopy and quantitative assays of p75 internalization in SCG cell bodies

Labeled cells were examined under a Zeiss LSM Pascal 5 (including a triple laser module Arg 458/488/514 nm, HeHe 543 nm, Carl Zeiss, Thornwood, NY) connected to an inverted microscope (AxioVert 2000) with a 63x objective. The ImageJ program was used to quantify the fluorescence intensity of the cells. Total cellular fluorescence was determined after subtracting the nonspecific fluorescence in images of untreated cells obtained with the same illumination and exposure conditions. Internalized MC192 or transferrin is presented as a ratio of the internalized receptor (intracellular fluorescence) to the cell surface-associated receptor (cell surface-associated fluorescence) or expressed as a percentage of intracellular fluorescence, with 100% set to the total cell-associated fluorescence (intracellular and surface-associated fluorescence). The absence of internalization or time 0 was defined as the fluorescence value obtained from cells treated with MC192 or transferrin at 4 °C. This value was subtracted from the internalization values obtained from cells treated at 37 °C. A previous study^24^ provides further details about the quantification procedures.

#### Electron microscopy (EM) analysis of the immunoendocytosis of p75

For EM assays, cultures of SCG neurons were starved with incubation medium. Then, the neurons were incubated with biotinylated MC192 conjugated with streptavidin Q-Dots (12 µg/mL) in incubation medium supplemented with 200 ng/mL BDNF (see reference^24^ for further experimental details). Cells were fixed by immersion in 3% glutaraldehyde, 0.05% picric acid, and 0.05 M cacodylate buffer, pH 7.4, for 2 hours. The cells were rinsed with the same buffer and immersed in 1% OsO_4_ for 30 min. Cells were dehydrated with a graded series of ethanol solutions and infiltrated with Epon. Ultrathin sections (50–80 nm) were stained with 2% uranyl acetate and lead citrate. Grids were examined with a Phillips Tecnai 12 electron microscope operated at 80 kV. Negative films were developed and scanned.

#### Metabolic inhibition assay

Axons of compartmentalized SCG cultures were incubated with the culture medium for SCGs supplemented with 12.5 mM KCl, 1 µg/mL blocking anti-NGF, 500 µM iodoacetic acid, 5 mM 2-deoxyglucose and 1 µM antimycin for 24 hours. Then, the culture was fixed, and immunofluorescence staining for βIII-tubulin was performed.

#### Immunoendocytosis of p75 and NRIF expression in SCGs infected with Rab5DN-GFP or EGFP adenoviruses

Neurons (6 DIV) were infected with adenoviruses driving the expression of Rab5DN-GFP or EGFP alone 24 hours after the initiation of the immunoendocytosis assay, as described below. Neurons were serum starved with incubation medium for 1 hour at 37 °C. Then, neurons were incubated wit MC192 (3 µg/mL) for 20 minutes at 4 °C; then, the media were replaced with media supplemented with 150 ng/mL of BDNF, and cultures were incubated at 37 °C for 90 minutes. Neuronal cultures were fixed and photographs of GFP and MC192 were captured using a spectral confocal microscope with a 60X objective (Nikon Eclipse C2).

SCGs (7 DIV) were first serum starved for 1 hour in incubation media supplemented with 12.5 mM KCl and then treated with BDNF (150 ng/mL) in incubation media supplemented with 12.5 mM KCl for 30 hours at 37 °C to visualize NRIF accumulation using immunofluorescence staining. After the treatment, cultures were fixed with 4% paraformaldehyde and permeabilized with 0.2% Triton and 5% fish gelatin in PBS for 1 hour at room temperature. Cultures were incubated with the anti-NRIF antibody (1:5000) overnight at 4 °C. After several washes with cold PBS, the fixed cultures were incubated with secondary antibodies conjugated to a fluorophore and Hoechst for 1 hour at room temperature, washed with PBS and mounted in Mowiol.

SCG neurons were treated as described above, NGF was eliminated with a blocking anti-NGF antibody (0,1 µg/mL) or treated with NGF (50 ng/mL) for 24 hours to visualize cleaved caspase 3 accumulation using immunofluorescence. After the treatment, cultures were treated as described above. Anti-cleaved caspase 3 and anti-βIII tubulin (1:1000) antibodies were incubated with the cultures overnight at 4 °C. After several washes with cold PBS, the fixed cultures were incubated with donkey anti-rabbit and -mouse secondary antibodies conjugated to Alexa Fluor 555 and Alexa Fluor 488 (1:300), respectively, for 1 hour at room temperature, washed with PBS and mounted in Mowiol. Neurons in which the fluorescence intensity of cleaved caspase 3 staining was three times greater than the baseline value (control neurons) and displaying positive signals for Rab5DN-GFP or EGFP were counted to quantify the percentage of neurons with cleaved caspase 3 staining.

#### Live cell imaging of p75 in compartmentalized SCG neurons treated with JNK and dynein inhibitors in the cell body compartment

The axonal compartment was serum starved with incubation medium for neurons supplemented with 12.5 mM KCl and 150 ng/mL TrkA Fc for 1 hour to measure the retrograde transport of p75 in compartmentalized SCG neuron cultures. A 200 nM MC192-biotin solution was mixed with 200 nM streptavidin-QDot 605 conjugates in DMEM on ice for 60 min to prepare MC192 conjugated to QDots 605 (MC192-QD). Then, the axons were incubated with MC192-QD and 150 ng/mL BDNF in SCG neuron incubation medium for 4 hours at 37 °C. The flux of the media was from cell bodies compartments to distal axons compartments to prevent the extracellular antibody and BDNF from gaining access to the cell bodies compartments (see Fig. 8a1). Thirty minutes prior to time-lapse imaging, each chamber was washed with incubation medium without phenol red to remove the MC192-QD and BDNF. Then, the cell body compartment was incubated with 10 µM SP600125 to inhibit JNK activity or 20 µM ciliobrevin D to inhibit dynein activity for 30 minutes at 37 °C. The flux of the media was reversed to prevent the drugs access to the distal axons compartments (see Fig. 8a2).

Movies were taken proximal to the microgrooves (see Fig. 8A) in the cell body compartment using an Olympus IX81 inverted spinning disk microscope with an objective of 40X. The microscope was equipped with an environmental chamber that maintained a constant temperature (37 °C) during live imaging. Time-lapse images were acquired at a speed of 1 frame/s for 2 minutes with a CCD Orca-R2 Hamamatsu C10600 camera. All data were processed and analyzed using NIH ImageJ 2.5 software.

### Statistics

For statistical analyses, the GraphPad Prism 7 program was used. Multiple comparisons were performed with one-way or two-way ANOVA and Tukey’s multiple comparisons test. A two-tailed Mann-Whitney test or unpaired Student’s t-test with Welch’s correction was applied to determine if two sets of data were significantly different. The data are presented as means ± SEM (standard errors of the means).

## Supplementary information

Movie 1 (noBDNF)

Movie 2 (BDNF)

Supplementary Information
