# Supplementary material for: c-Jun N-terminal kinase (JNK)-dependent internalization and Rab5-dependent endocytic sorting mediate long-distance retrograde neuronal death induced by axonal BDNF-p75 signaling
Source: Sci Rep. 2019 Apr 15;9:6070. doi: 10.1038/s41598-019-42420-6 (PMC6465280; doi:10.1038/s41598-019-42420-6)
Supplement: Supplementary file 3 — Supplementary Information [file 41598_2019_42420_MOESM3_ESM.pdf]

c-Jun N-terminal kinase (JNK)-dependent internalization and Rab5-dependent endocytic sorting mediate long-distance retrograde neuronal death induced by axonal BDNF-p75 signaling.

Escudero CA[1#], Cabeza C[1#&] Moya-Alvarado G[1], Maloney MT[2], Flores CM[1], Wu C[4], Court FA[3], Mobley WC[4], and Bronfman FC[1*].

[1]Center for Aging and Regeneration (CARE UC) and Department of Physiology, Faculty of Biological Sciences. Pontificia Universidad Católica de Chile, Santiago, Chile;
[2]Department of Neurology and Neurological Sciences, Stanford University School of Medicine, Stanford, CA, USA;
[3]Center for Integrative Biology. Faculty of Sciences. Universidad Mayor, Santiago, Chile and FONDAP Center for Geroscience, Brain Health and Metabolism, Santiago, Chile;
[4]Department of Neurosciences, University of California, San Diego, La Jolla, California 92093, USA

# Both authors contributed equally to this work.

& Current address: Center of Cellular and Integrative Physiology, Faculty of Medicine, Clínica Alemana, Universidad del Desarrollo, Santiago, Chile.

*Corresponding author, *fbronfman@bio.puc.cl*

Legend movie 1 and movie 2. p75 retrograde transport is increased by BDNF.
Compartmentalized sympathetic neuron cultures were treated with MC192-Qdots labelling p75 in the extracellular domain for 4 hours at 37ºC in the axonal chamber before imaging in the absence (movie 1) or presence (movie 2) of BDNF (150 μg/mL). After, movement of vesicles were observed using real-time microscopy as described in the material and methods sections.

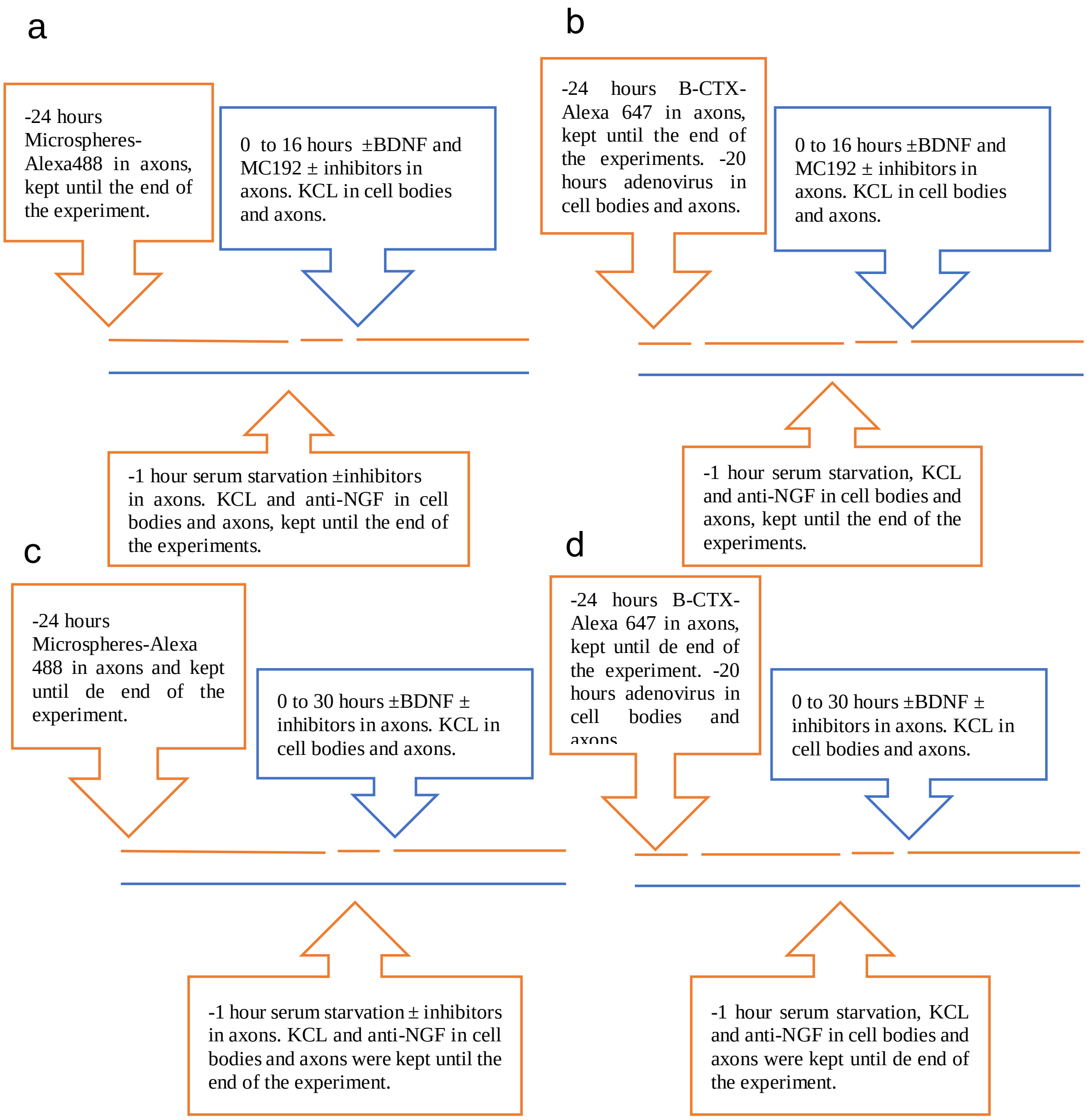

Supplementary Figure S1. The scheme summarizes the design of the experiment used to measure the retrograde transport of p75 **(a** and **b)** and apoptosis upon the addition of BDNF to axons **(c** and **d)** in compartmentalized SCGs cultures. Time 0 was when BDNF was added to axons. In **(a)** and **(c)**, axons were treated with JNK and dynein inhibitors, and in **(b)** and **(d)**, neurons were transduced with adenoviruses driving the expression of GFP or a dominant negative mutant of Rab5 (Rab5DNGFP). The blue line indicates time course of the experiment. The red line is broken each time a new reagent was added to cell bodies or axons. Blue boxes indicate the moment when BDNF was added to axons. In the experiment without BDNF, the anti-BDNF antibody was added to axons. Red boxes indicate the application of different treatments before the addition of BDNF. See the materials and methods section for the concentration of each reagent applied.

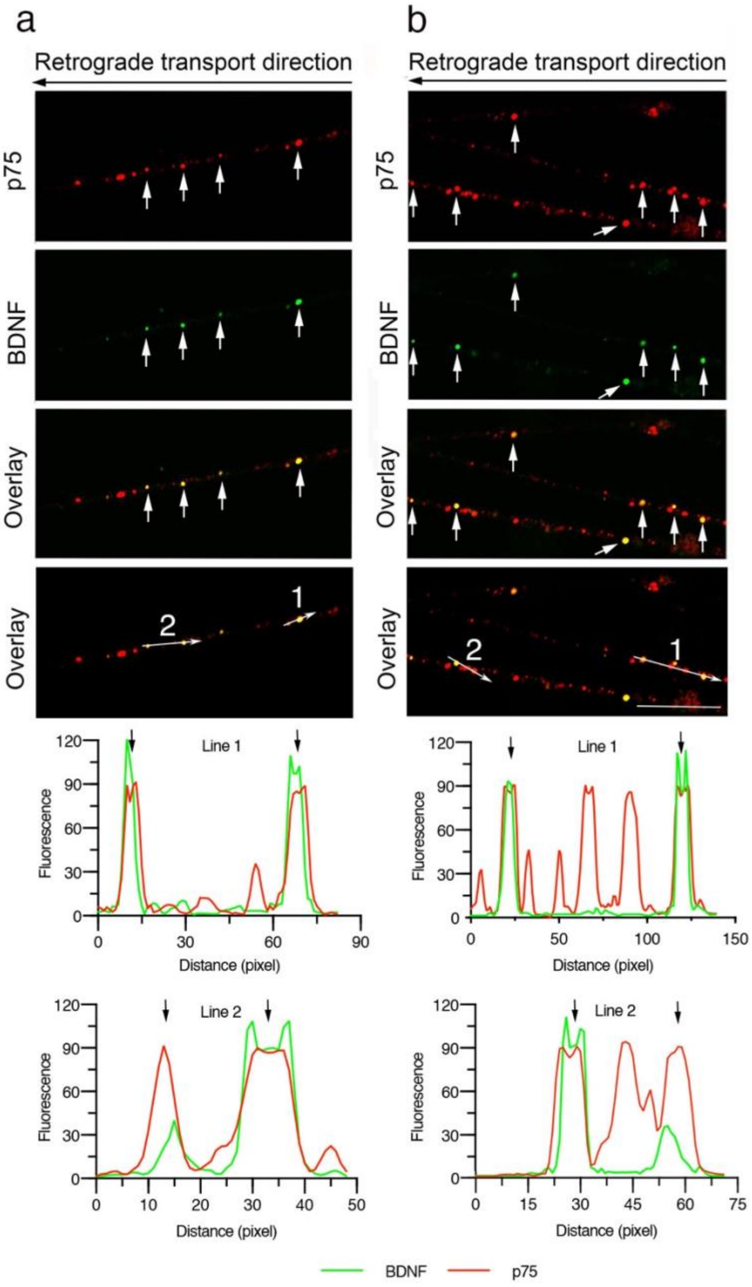
a
b
Retrograde transport direction
Retrograde transport direction
p75
BDNF
Overlay
Overlay
1
2
Line 1
Line 2
Fluorescence
Distance (pixel)
BDNF
p75

Supplementary Figure S2. p75 and BDNF colocalization in axons. Confocal microscopy images of axons from compartmentalized sympathetic cultures treated with BDNF and MC192-Alexa Fluor 594 (to label p75) for 4 hours at 37°C in the axonal chamber. **(a)** and **(b)** Images of axons from different cultures receiving the same treatment. After fixation, BDNF was labeled with a rabbit polyclonal antibody against BDNF, followed by detection with a mouse secondary antibody (green). The images are representative of 4 different compartmentalized cultures. Arrows indicate vesicles containing both p75 and BDNF. Lower panels depict graphs showing the pixel by pixel fluorescence intensity profile. A green line indicates the fluorescent profile of the BDNF-associated fluorescence and the red lines indicate the fluorescent profile of the p75-associated fluorescence at the white lines with arrows drawn in the overlay in the bottom of the panel. The white line shows where pixels were examined for content of BDNF, p75, or both; the arrowheads mark the direction (left-to-right) for reporting pixel data recorded along the white lines.

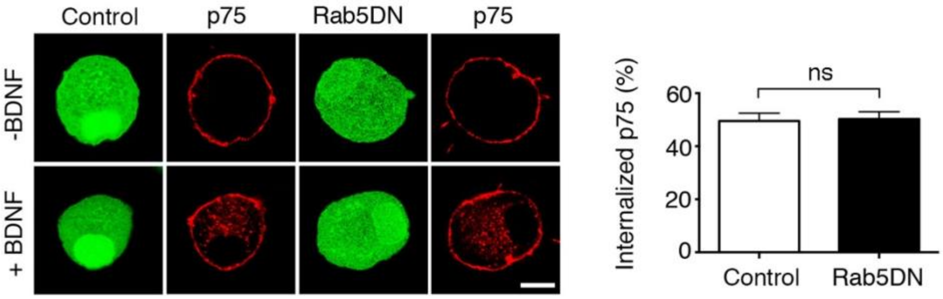

Supplementary Figure S3. Rab5 is not required for p75 internalization. Confocal microscopy images of noncompartmentalized SCG neurons infected with an adenovirus driving the expression of GFP or Rab5DN-GFP (green). Live neurons were incubated with MC192 to label p75 in the membrane and treated with BDNF (150 ng/mL) for 90 minutes to induce p75 endocytosis. After fixation, neurons were incubated with a mouse secondary antibody conjugated to Alexa Fluor 555 (red) to visualize MC192. The internalized p75 protein was normalized to levels of the membrane protein under control conditions. Scale bar, 10 µm. Twenty neurons from 2 different experiments were quantified at each time point. Statistically significant differences were analyzed using two-tailed unpaired t-tests with Welch’s correction. p=0.86.

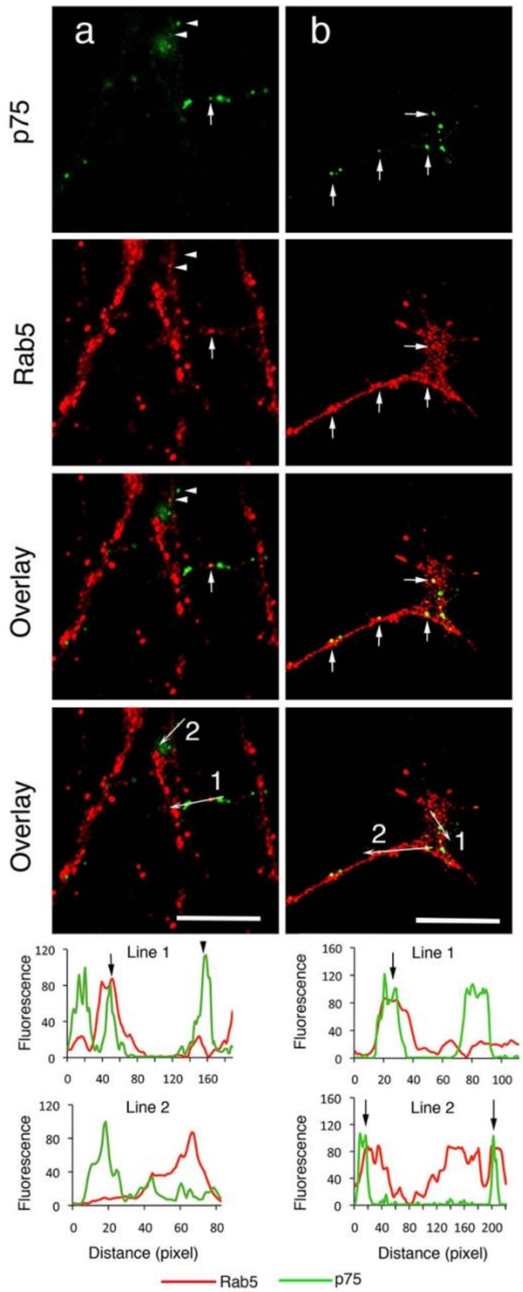

Supplementary Figure S4. Endocytosed p75 partially colocalizes with endogenous Rab5 in the axons (a) and growth cones (b) of sympathetic neurons. At 6 DIV, the axon compartment of compartmentalized sympathetic neurons was treated with MC192-Alexa Fluor 594 (green, to label p75) and BDNF for 4 hours at 37°C. After fixation, the axons were immunostained for Rab5 (red). Scale bar, 10 µm. Arrows indicate vesicles containing both p75 and Rab5. Arrowheads indicate p75- or rab5-positive vesicles that were not colocalized. Lower panels depict graphs showing the pixel by pixel fluorescence intensity profile. A green line indicates the fluorescent profile of the p75-associated fluorescence and the red lines indicate the fluorescent profile of the Rab5-associated fluorescence of the white lines with arrows drawn in the overlay in the bottom of the panel. The arrowheads indicate the direction of the line, where 0 pixels is when the line starts, and the arrowhead depicts the termination of the line.

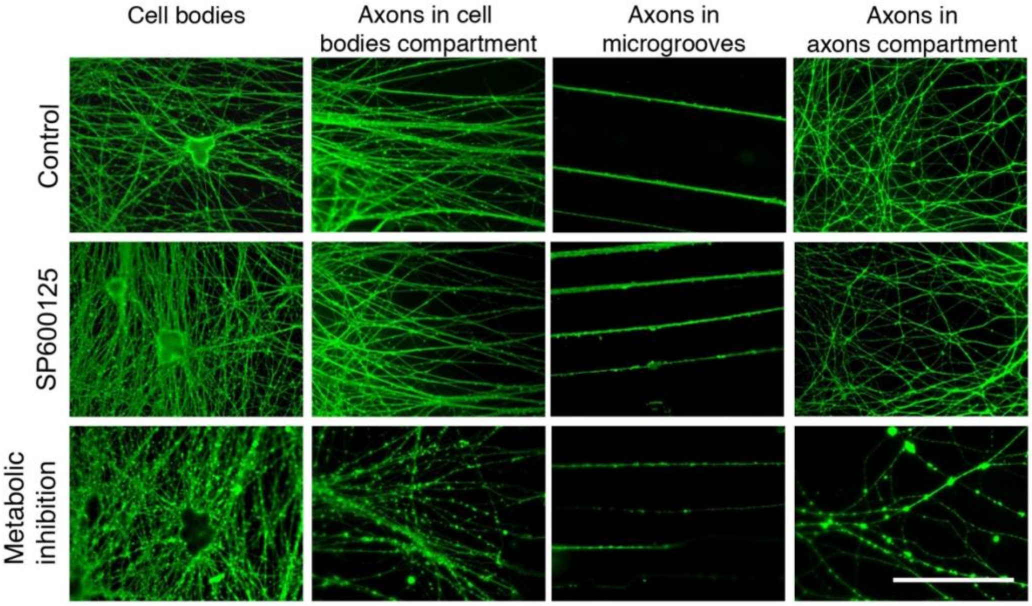

Supplementary Figure S5. SP600125, a JNK inhibitor, does not affect the axonal integrity of sympathetic neurons. Fluorescence microscopy images of 6 DIV compartmentalized sympathetic neurons in which the axonal chamber was treated with SP600125 (10 μM) for 30 hours and fixed. After fixation, the axons were immunostained with an antibody against βIII-tubulin to examine axonal integrity. Metabolic inhibition was used as a positive control for axonal degeneration. The SP600125 treatment did not affect the axonal integrity, in contrast to axons treated with metabolic inhibitors. Scale bar, 100 μM.

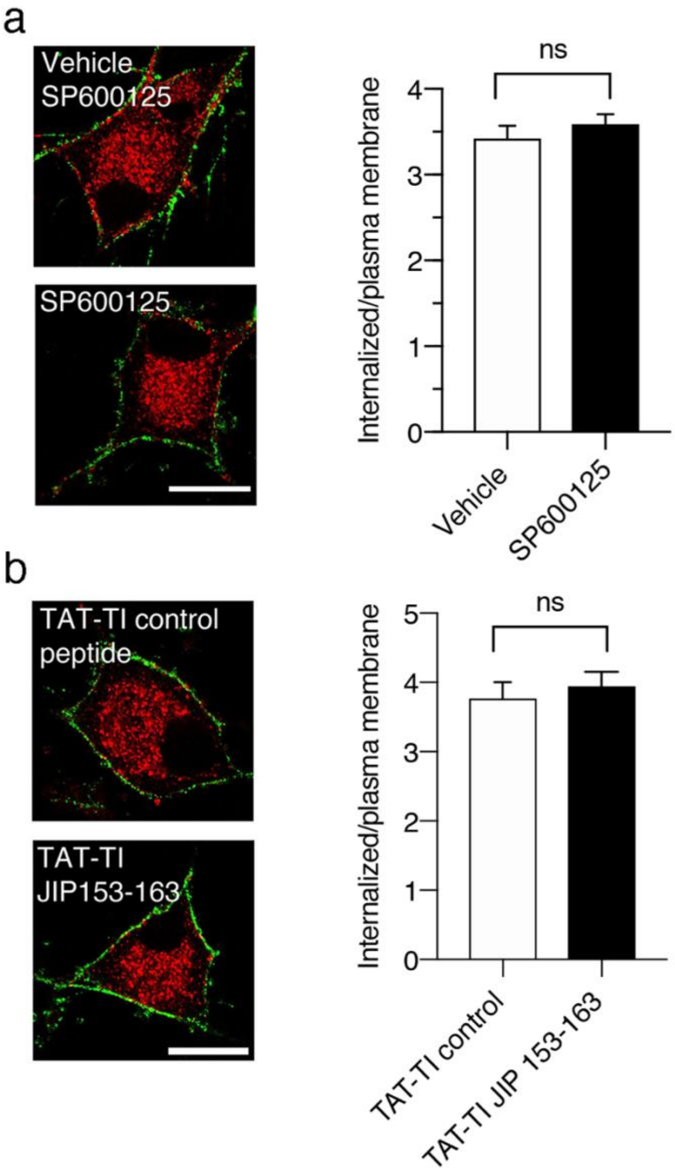

Supplementary Figure S6. JNK does not participate in transferrin internalization in SCG neurons. Confocal microscopy images of sympathetic neurons treated with transferrin-Alexa Fluor 594 (red) for 60 min at 37°C in the absence (vehicle and TAT-TI control peptide) or presence of the JNK inhibitor SP600125 **(a)** and TAT-TI-JIP 153-163 peptide **(b)**, as indicated in Figure 7. Green labeling indicates cell surface p75 labeled with MC192-Alexa Fluor 488. Scale bar, 10 μm. The bottom panel shows the levels of internalized transferrin after different treatments (relative fluorescence/normalized to cell surface, p75). Forty cells from 2 independent experiments were quantified.

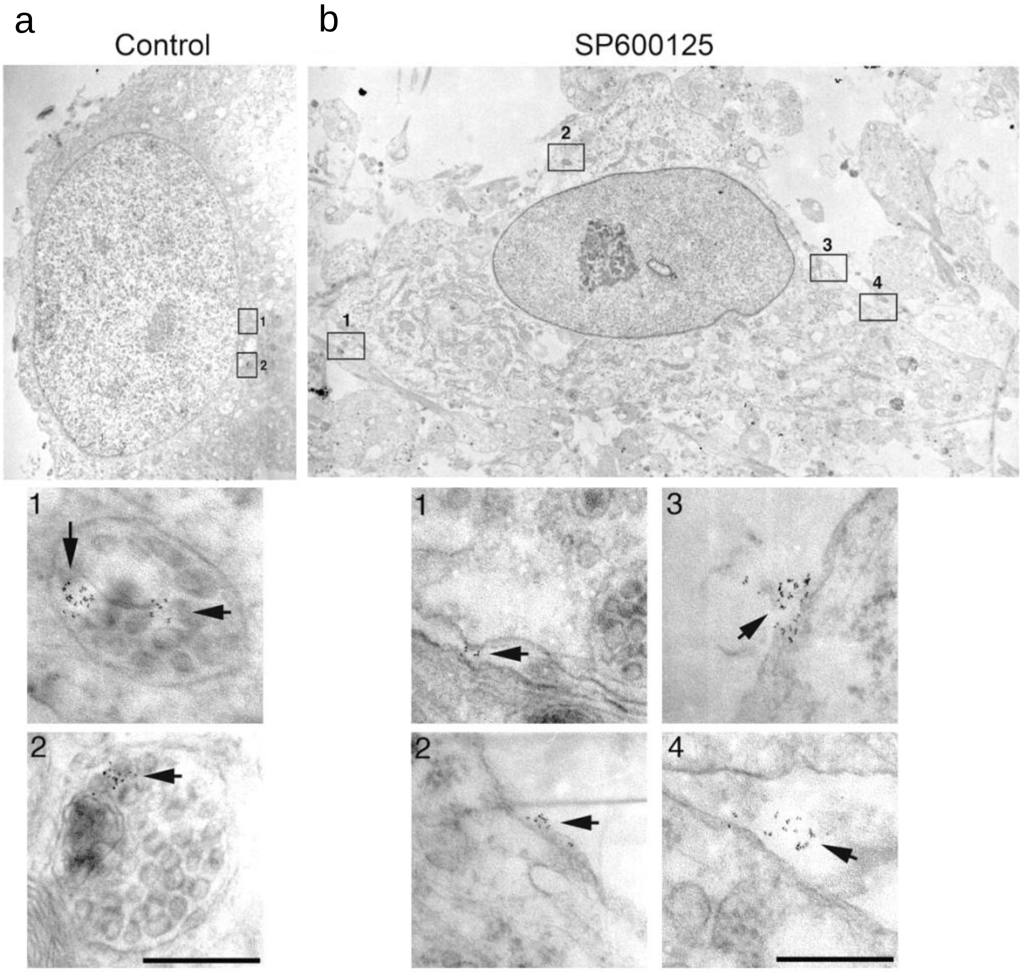

Supplementary Figure S7. Electron microscopy analysis of the effect of SP600125 on p75 internalization. (a) Electron microscopy images of sympathetic neurons treated with BDNF and MC192Q-dots for 240 min at 37°C. The squares indicate locations in the cell that are magnified in the bottom panels. (**b)** Electron microscopy images of sympathetic neurons treated with BDNF and MC192Q-dots for 240 min at 37°C in the presence of SP600125. The squares indicate locations in the cell that are magnified in the bottom panels. Scale bar, 300 nm. The arrows indicate labeled p75. In the presence of SP600125, p75 is observed outside the cell.
